# Is Butter Back? A Systematic Review and Meta-Analysis of Butter Consumption and Risk of Cardiovascular Disease, Diabetes, and Total Mortality

**DOI:** 10.1371/journal.pone.0158118

**Published:** 2016-06-29

**Authors:** Laura Pimpin, Jason H. Y. Wu, Hila Haskelberg, Liana Del Gobbo, Dariush Mozaffarian

**Affiliations:** 1 Friedman School of Nutrition Science & Policy, Tufts University, 150 Harrison Avenue, Boston, MA, United States of America; 2 The George Institute for Global Health, University of Sydney, Sydney, Australia; 3 Cardiovascular Medicine, Stanford School of Medicine, Palo Alto, CA, United States of America; Hunter College, UNITED STATES

## Abstract

**Background:**

Dietary guidelines recommend avoiding foods high in saturated fat. Yet, emerging evidence suggests cardiometabolic benefits of dairy products and dairy fat. Evidence on the role of butter, with high saturated dairy fat content, for total mortality, cardiovascular disease, and type 2 diabetes remains unclear. We aimed to systematically review and meta-analyze the association of butter consumption with all-cause mortality, cardiovascular disease, and diabetes in general populations.

**Methods and Findings:**

We searched 9 databases from inception to May 2015 without restriction on setting, or language, using keywords related to butter consumption and cardiometabolic outcomes. Prospective cohorts or randomized clinical trials providing estimates of effects of butter intake on mortality, cardiovascular disease including coronary heart disease and stroke, or diabetes in adult populations were included. One investigator screened titles and abstracts; and two reviewed full-text articles independently in duplicate, and extracted study and participant characteristics, exposure and outcome definitions and assessment methods, analysis methods, and adjusted effects and associated uncertainty, all independently in duplicate. Study quality was evaluated by a modified Newcastle-Ottawa score. Random and fixed effects meta-analysis pooled findings, with heterogeneity assessed using the I^2^ statistic and publication bias by Egger’s test and visual inspection of funnel plots. We identified 9 publications including 15 country-specific cohorts, together reporting on 636,151 unique participants with 6.5 million person-years of follow-up and including 28,271 total deaths, 9,783 cases of incident cardiovascular disease, and 23,954 cases of incident diabetes. No RCTs were identified. Butter consumption was weakly associated with all-cause mortality (N = 9 country-specific cohorts; per 14g(1 tablespoon)/day: RR = 1.01, 95%CI = 1.00, 1.03, P = 0.045); was not significantly associated with any cardiovascular disease (N = 4; RR = 1.00, 95%CI = 0.98, 1.02; P = 0.704), coronary heart disease (N = 3; RR = 0.99, 95%CI = 0.96, 1.03; P = 0.537), or stroke (N = 3; RR = 1.01, 95%CI = 0.98, 1.03; P = 0.737), and was inversely associated with incidence of diabetes (N = 11; RR = 0.96, 95%CI = 0.93, 0.99; P = 0.021). We did not identify evidence for heterogeneity nor publication bias.

**Conclusions:**

This systematic review and meta-analysis suggests relatively small or neutral overall associations of butter with mortality, CVD, and diabetes. These findings do not support a need for major emphasis in dietary guidelines on either increasing or decreasing butter consumption, in comparison to other better established dietary priorities; while also highlighting the need for additional investigation of health and metabolic effects of butter and dairy fat.

## Introduction

Growing uncertainty and changing views on the role of butter in cardiovascular disease (CVD) have been prominently discussed, including in the New York Times and Time Magazine. [1, 2] This has partly arisen from increasing controversy on the utility of focusing on isolated macronutrients, such as saturated fat, for determining risk of chronic diseases. Mounting evidence indicates a need to shift away from isolated macronutrients toward food-based paradigms for investigating dietary priorities for chronic diseases. [3, 4] The 2015 Dietary Guidelines Advisory Committee (DGAC) recommended replacing animal fats, including butter, with non-hydrogenated vegetable oils high in unsaturated fats and relatively low in saturated fatty acids. [4] Yet, the DGAC also concluded that further research was needed on the effects of saturated fat from different food sources, including animal products, on cardiovascular risk, because different food sources contain varying specific fatty acid profiles as well as other constituents that may result in distinct lipid and metabolic effects. [4]

For example, growing evidence supports potential metabolic benefits of certain dairy products, such as yogurt and possibly cheese, on risk of type 2 diabetes [5, 6], which may even relate to benefits of dairy fat. [7–9] However, the relationship of butter, which is highest in dairy fat, with diabetes remains unclear. The long-term effects of butter consumption on other major endpoints, such as all-cause mortality and CVD, are also not well-established. Previous reviews have evaluated only some of these outcomes, included butter as part of a wider investigation into dairy foods or types of fats [10–12], and utilized methods that provided imprecise estimates of effect, precluded dose-response evaluation, or may have introduced unintended bias (e.g., due to inclusion of crude, unadjusted effect estimates).

A systematic review of the evidence for of the relationship between butter consumption and long-term health is of considerable importance, both for understanding food-based health as well as informing dietary recommendations for clinicians and policy makers. The US Department of Agriculture has documented a 40-year record high in US butter consumption in 2014 [13], making a synthesis of the evidence on butter and major chronic diseases highly relevant and timely.

To synthesize the evidence on the long-term association of butter consumption with major health endpoints, we conducted a systematic review and meta-analysis of prospective observational studies or randomized clinical trials investigating butter consumption and all-cause mortality, CVD including coronary heart disease (CHD) and stroke, and type 2 diabetes in general populations.

## Materials and Methods

We followed Meta-analysis of Observational Studies in Epidemiology (MOOSE) guidelines (S1 File) for observational studies and Preferred Reporting Items for Systematic Reviews and Meta-Analyses (PRISMA) (S2 File) guidelines for trials during all stages of design, implementation, and reporting.

### Data sources

We performed a systematic search for all prospective cohort studies and randomized clinical trials examining butter consumption and all-cause mortality, CVD including CHD and stroke, or type 2 diabetes. Electronic searches were performed using PubMed (www.ncbi.nlm.nih.gov/pubmed), EMBASE (www.scopus.com), The Cochrane Library (www.cochranelibrary.com), Web of Knowledge (www.webofscience.com*)*, CAB Abstracts and Global Health (www.ovid.com), CINAHL (www.ebscohost.com) and grey literature searches of SIGLE (www.opengrey.eu) and ZETOC (www.zetoc.mimas.ac.uk/) from the earliest indexing year of each database through May 2015, without language or other restrictions. Search terms included butter, margarine, dairy, dairy products, yogurt, cheese, ghee, animal fat, solid fat, cardiovascular diseases, heart disease, stroke, myocardial infarction, heart attack, cerebrovascular disease, cerebrovascular accident, sudden death, diabetes, mortality and deaths; see S3 File for a full listing. For all final included articles, we further performed hand-searches of citation lists and a review of the first 20 related references on PubMed for additional eligible reports.

In addition, among studies excluded by title and abstract screening, several were identified evaluating overall dietary patterns (e.g., Mediterranean, Western, etc.) To ensure that we were not missing effect estimates for butter contained within these reports (e.g., in supplementary tables on the individual components of these dietary patterns), we also reviewed the full texts of the first 15 identified studies of dietary patterns. None of these studies reported individual effect estimates for butter, so further diet pattern studies lacking any information on butter in the title or abstract were excluded

### Study selection

Titles and abstracts of all identified eligible articles were screened by one investigator. For all potentially relevant articles, the full text was retrieved and reviewed independently and in duplicate by two reviewers according to the eligibility criteria.

We searched for all randomized controlled trials or prospective cohorts (cohort, nested case-subcohort, nested case-control) conducted in adults (18+ y) that provided a multivariate-adjusted effect estimate (or unadjusted effect estimate in trials) and measure of statistical uncertainty of the relationship between total or added butter and all-cause mortality, incident CVD including CHD or stroke, and incident diabetes. We excluded animal, ecologic, quasi-experimental, and non-prospective observational studies (case reports, cross-sectional studies, and retrospective case-control studies), editorials, letters, and reviews (S4 File).

Studies were also excluded if evaluating only children or populations with major end-stage diseases such as cancer; if duration of intervention or follow-up was less than 3 months; if consumption of butter was not separately distinguishable from other dairy product or fats; if evaluating only soft endpoints (e.g. angina pectoris, coronary insufficiency); or, for observational studies, if providing only unadjusted (crude) effect estimates. When duplicate publications were identified, the report including the largest number of cases for each endpoint of interest was selected. If references were only available in abstract form (e.g. from meeting proceedings or conference presentations), data were extracted if sufficient detail was available; if not, a relevant publication was searched for in PubMed.

### Data extraction

Data from the included studies were independently extracted in duplicate by two investigators using a standardized and piloted electronic form (Microsoft Excel). Any differences in extraction were resolved by consensus. Information was extracted on the publication (first author name, contact information, publication year), study details (name, location, design), population (age, gender, race, socioeconomic status, body mass index), sample size, dates of recruitment, duration of follow-up, dietary assessment (dates, method, definition, categories), outcome(s) (assessment method, definition), covariates and analysis methods, and multivariate-adjusted effect estimates and associated uncertainty. To evaluate dose-response, we extracted continuous effect estimates when available; and for categorical analyses, collected additional information on median exposure, number of participants or person-years, and number of events in each category. Missing information in any category was obtained by direct author contact or, if necessary, estimated using a standard approach (see S5 File).

When more than one multivariable model was reported, we used the risk estimate including the greatest number of potential confounders but not potential mediators (e.g., blood cholesterol). If the main multivariable model included covariates which could either be confounders or intermediates, this was utilized rather than a model with crude or minimal covariate adjustment. When energy intake was included as a covariate, body mass index was not considered to be an intermediate variable, so models adjusting for body mass index were extracted (this only arose in one study, by Buijsse *et al*. [14]). The effect results from the Guasch-Ferre *et al*. [15] study were estimated using the models of risk of diabetes associated with substitution of olive oil for equivalent amounts of butter, and our results were confirmed and validated by contact with the authors.

### Quality assessment

We adapted the Newcastle-Ottawa quality scale(NOS) [16] to assess study quality, based on five criteria evaluating the reporting and appropriateness/representativeness of participant inclusion and exclusion criteria (combining the first two items of the NOS Selection scale), participant attrition (NOS adequacy of follow-up item), control for confounding (NOS Comparability scale), assessment of exposure (NOS ascertainment of exposure item), and assessment of outcome (combining the first two items of the NOS Outcome scale). One point was allocated per criterion met, the sum of which provided an overall quality score. A score between 0 and 3 was considered low-quality; and 4 to 5, high-quality. Quality scores were assessed independently and in duplicate by two investigators, with any differences resolved by consensus.

### Data synthesis and statistical analysis

Reported hazard ratios were assumed to approximate relative risks (RRs). We used the two-stage generalized least-squares trend estimation method described by Greenland and Longnecker [17, 18] to perform dose-response analysis and compute study-specific linear estimates and 95% CIs across categories of butter intake. Butter intakes across studies were standardized at the study level to 14 g/d, corresponding to one United Stated Department of Agriculture-defined serving. [19] Study-specific dose-response estimates were then pooled to derive an overall estimate using inverse-variance weighted DerSimonian and Laird meta-analysis with random effects. [20] Because random effects can result in larger weights for small outlier studies, we also conducted fixed effects meta-analysis for comparison. For reports presenting results only by study subgroups (e.g., men, women), we first pooled the study-specific subgroups using fixed-effect meta-analysis to obtain a single estimate per study.

Heterogeneity between studies was quantified using the I^2^ statistic, with statistical significance (P<0.05) evaluated by the Q statistic. [21] We considered I2 values between 25% and 50%, between 50% and 75% and above75% as upper thresholds for low, moderate, and high heterogeneity, respectively. We planned pre-specified subgroup analyses to further explore potential heterogeneity in results by gender, population mean age and body mass index, duration of follow-up, and study quality score. Restricted cubic spline models [22] with knots at the 25th, 50th, and 75th percentiles were used to examine potential nonlinear relations.

Potential for publication bias was assessed by visual inspection of funnel plots and by Egger’s test. [23] We used Duval and Tweedie’s non-parametric “trim and fill method” to adjust the pooled estimates for any hypothetically missing studies. [24] All analyses were conducted using Stata 13 (StataCorp, College Station, Texas), with 2-tailed alpha = 0.05.

## Results

### Study characteristics

**Fig 1:**
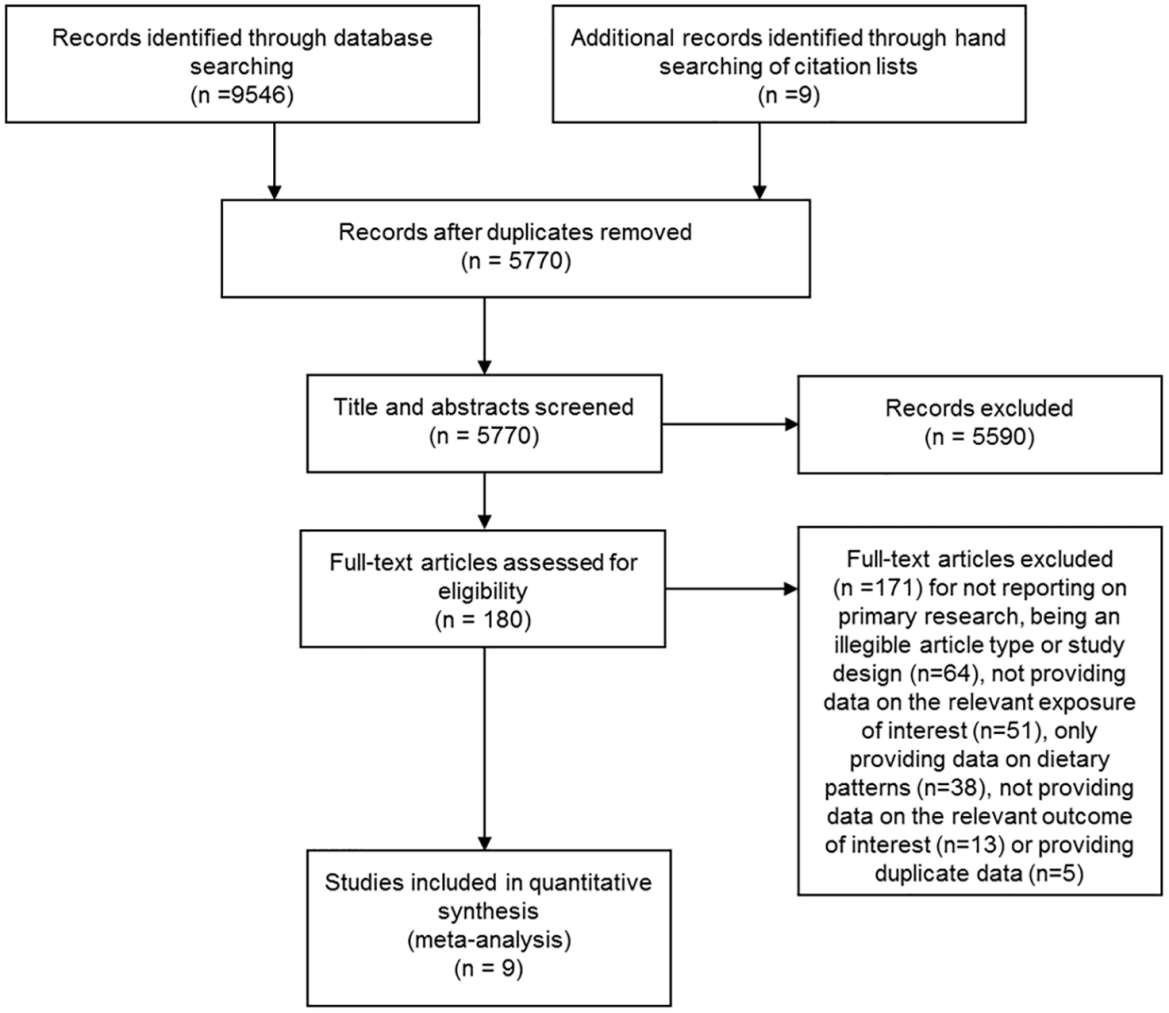
Flowchart of study identification and selection process. All systematic review and meta-analyses methods conducted according to PRISMA guidelines.

Among 5,770 unique abstracts, we identified 9 publications including 15 country-specific cohorts (Fig 1), together reporting on over 636,000 unique participants with 6.5 million person-years of follow-up and including 28,271 total deaths, 9,783 cases of incident CVD, and 23,954 cases of incident diabetes (Table 1). No randomized clinical trials evaluating butter and these endpoints were identified. Outcomes in each study were generally assessed by review of medical records, linkage to death certificates, or hospital registers.

**Table 1:** Characteristics of 9 prospective studies providing risk estimates for the association between butter intake and total mortality, CVD and type 2 diabetes.

Author (year)	Country	Population source and age	Diet assessment	Type of exposure	Median intake (top category (g/d))	Disease ascertainment	Disease outcome	Sample size	Mean follow-up (y)$	N events	Person- years	RR (95%CI)	Covariate adjustment	Quality score
**Avalos (2012)** [25]	United States	Residents of Californian community; (mean 70.6 yrs. (SD 9.8))	FFQ	Total butter	7.0	Questionnaires at follow-up visits and annual mailed surveys.	CHD	1,759	16.2	451	28,496	0.97 (0.59, 1.61)	Age, BMI, diabetes, hypertension, LDL-cholesterol, current estrogen use.(in women only)	5
**Buijsse (2015)** [14]	Multi-country (EPIC)	General population participants of the 8 European countries in EPIC InterAct case-cohort study; (mean 52 yrs.).	FFQ and food records	Total butter	18.7	Self-report, record linkage to health registers, pharmacy database and hospital admissions or mortality data & confirmation from another independent source for participants with <2 sources.	Diabetes	25307	12.3	11,059	183,761	0.94 (0.90, 0.98)	Study center, energy intake, alcohol intake, gender, education, smoking status, physical activity, BMI	5
**Ericson (2015)** [26]	Sweden	Women born between 1923–1950 and all men born 1923–1945 living in the city of Malmo (mean 58 yrs.)	FFQ and food records	Total butter & butter blends	33.0	Registry follow-up at screenings or interviews at follow-up.	Diabetes	26,930	14.0$	2,860	377,642	0.93 (0.89, 0.98)	Age, gender, education, method version, season, total energy intake, physical activity, smoking, alcohol intake, and BMI.	5
**Goldbohm (2011)** [27]	Netherlands	Subjects 55–69 yrs., from the general population sampled from municipal population registries.	FFQ				All-cause mortality	120,852	10.0$	16,136	1,044,757	1.02 (1.00, 1.03)	Age, education, smoking, physical activity, multivitamin use, alcohol use, energy, e-adjusted MUFA and PUFA intake and fruit and vegetable consumption	5
							CHD	120,852	10.0$	2,689	1,044,757	1.03 (0.95, 1.11)		
							Stroke	120,852	10.0$	842	1,044,757	0.99 (0.88, 1.10)		
**Guasch-Ferre (2015)** [15]	United States	Women from NHS & NHSII (26–65 years) Mean 44 yrs.	FFQ	Total butter	NHS:11.4NHS II:9.8	Validated supplementary questionnaire on symptoms, plasma glucose concentrations and treatment/medication for diabetes	Diabetes	145,087	22$	9,652	1,595,957	0.98 (0.94, 1.02)	Age, ethnicity, ancestry, smoking status, alcohol intake, dietary pattern (Alternative Healthy Eating Index score, and total energy intake, physical activity, family history of diabetes, history of hypertension, hypercholesterolemia, multivitamin use, postmenopausal status and menopausal hormone use.	5
**Larsson (2009)** [28]	Finland	Men who resided in southwestern Finland and smoked ≥5 cigarettes per day at baseline (mean 58 yrs.).	FFQ	Total butter	79.0	Record linkage with National Register of Causes of Death using ICD classification	Cerebral infarction	26,556	13.6		360,187	1.00 (0.98, 1.02)	Age, supplementation group, education, intakes of total energy, alcohol, caffeine, sugar, red meat, poultry, fish, fruit, fruit juices, vegetables, potatoes, whole grains, and refined grains, smoking, BMI, serum total cholesterol, serum HDL cholesterol, histories of diabetes and heart disease, leisure-time physical activity	4
							Subarachnoid hemorrhage	26,556	13.6	2,702	360,187	0.99 (0.91, 1.08)		4
							Intracerebral hemorrhage	26,556	13.6	196	360,187	1.04 (0.98, 1.11)		
**Montonen (2005)** [29]	Finland	Finish citizens aged≥15yrs participating in the Mobile Clinical Health Examination Survey (mean 52 yrs.)	Diet history interview	Total butter	67.5	Record linking to nationwide social insurance register of diabetic treatment drug reimbursement.	Diabetes	4,304	23.0	383	84,328	1.04 (0.94, 1.15)	Age, gender, geographic area, energy intake, smoking, family history or diabetes, BMI,	4
**Sluik (2014)** [30]	Multi country (EPIC)	General population participants of the 8 European countries in EPIC (mean 52 yrs.).	FFQ and food records	Total butter & margarine **	33.0*	Record linkage with cancer or death registries, boards of health. In DE, follow-up mailings to participants and next of kin and inquiries to municipality registries, and regional health services.	All-cause mortality	258,911	9.9	12,135	2,552,218	1.01 (0.99, 1.03)	Age and center-stratified, adjusted for gender, education, underlying dietary patterns, alcohol consumption, smoking, physical activity and prevalence of heart disease, cancer or stroke,	4
**Sonestedt(2011)** [31]	Sweden	Women born 1923–1950 and men born 1923–1945 living in the city of Malmo (mean 57 yrs.).	FFQ and food records	Total butter	49.0	Linkage to Hospital Discharge and cause-of-death Registers and local stroke Register in Malmo.	CHD	26,445	13.0	1,344	312,476	0.98 (0.94, 1.02)	Age, gender, season, method, education, energy intake, intake of vegetables, fruit and berries, fish and shellfish, meat, coffee and whole grains, BMI, smoking, alcohol consumption, leisure-time physical activity,.	4
							Stroke	26,445	13.0	1,176	312,476	0.99 (0.94, 1.04)		

No RCTs were identified. Total participants N = 636,151; Total person-years = 6,539,822; Total events N = 62,008;

CHD: Coronary heart disease; CVD: Cardiovascular disease; DE: Germany; EPIC: European Prospective Investigation into Cancer and Nutrition; FFQ: Food-frequency questionnaire; ICD: International Classification of Diseases; IHD: Ischemic Heart Disease; NHS & NHSII: Nurses’ Health Study I and II;

*75^th^ percentile of intake (not presented categorically);

** Exposure combined butter and margarine, author contact clarified that margarine intake was low in this population.

^$^ Maximum follow-up reported: Studies obtaining a quality score of 4 were primarily due to lack of reporting on loss to follow-up.

Avalos et al. and Goldbohm et al. reported results separately for men and women; Buijsse et al. reported results separately for diabetics and non-diabetics; Person-years for Buijsse et al. and Montonen et al. were estimated.

Diet was generally assessed by detailed, semi-quantitative food frequency questionnaires; one cohort utilized a structured diet history interview (Table 1). The median butter consumption across studies ranged from 4.5g/d (0.3 servings/d) in the European Prospective Investigation into Cancer and Nutrition (EPIC) studies to 46 g/d (3.2 servings/d) in Finland. Mean participant age ranged from 44 to 71 years. All studies were published between 2005 and 2015, and included 1 in the Netherlands, 2 in the US, 2 in Finland, 2 in Sweden, and 2 from the multi-country, multi-cohort EPIC study which included 8 country-specific cohorts from Denmark, France, Italy, Germany, the Netherlands, Spain, Sweden and the UK. Five of the studies presented results from models with optimal covariate adjustment including demographics, clinical risk factors, and other dietary habits; the remainder provided results with moderate covariate adjustment.

### All-cause mortality

**Fig 2:**
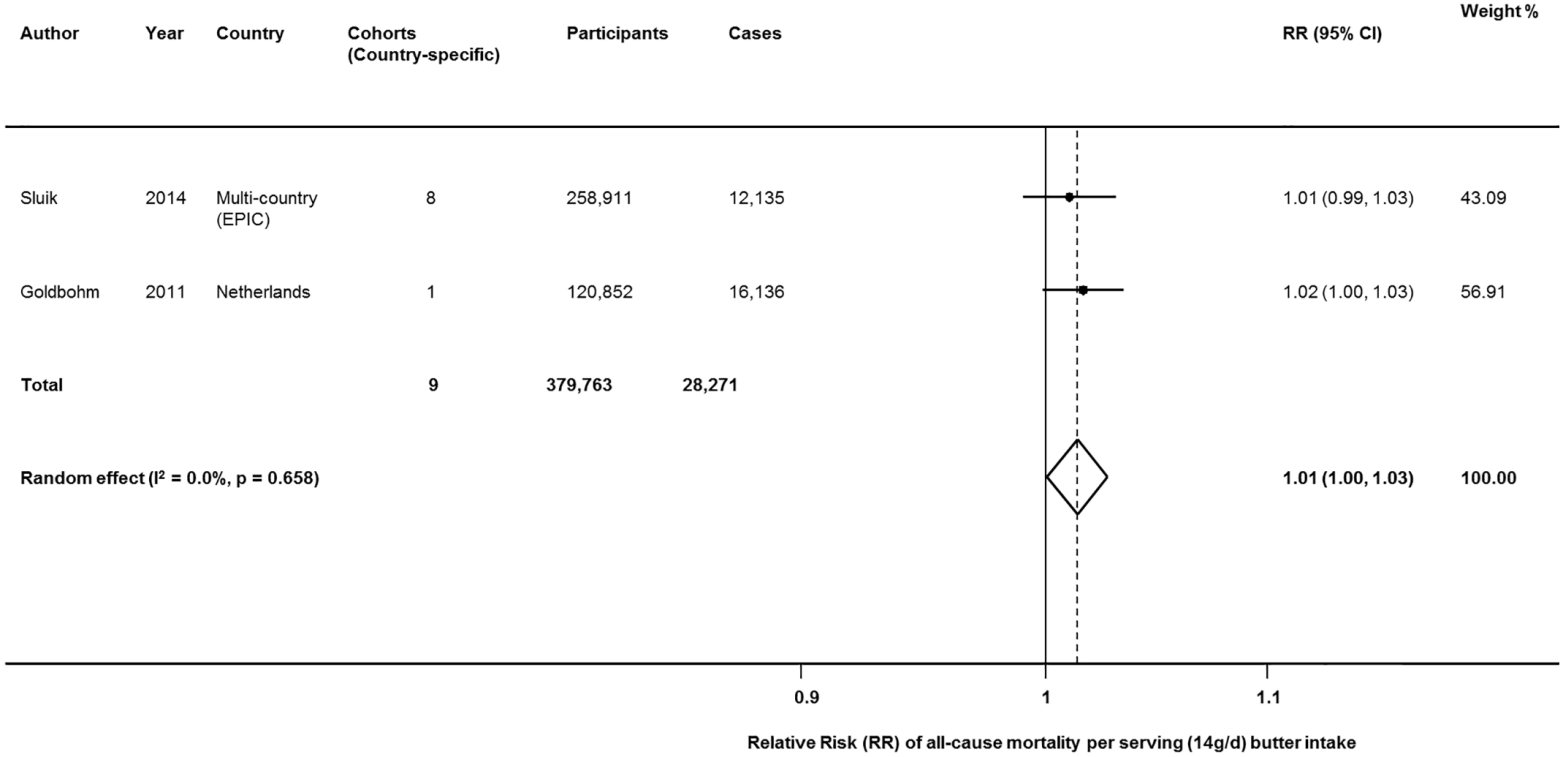
Butter consumption and risk of all-cause mortality. Within-study dose-response RRs were derived from reported linear effects or generalized least-squares trend estimation for studies reporting categories of intake, and pooled using both inverse-variance weighted random and fixed effects meta-analysis.

Two large studies including 9 country-specific cohorts evaluated butter intake and all-cause mortality, including 379,763 participants and a total of 28,271 deaths. [27, 30] Pooling these studies, each daily serving of butter (14g/d) was associated with a 1% higher risk of death (RR = 1.0134 (95%CI = 1.0003, 1.0266; P = 0.045) (Fig 2). No significant heterogeneity was identified (I^2^ = 0.0%). Findings were similar when explored using fixed-effects (P = 0.045).

### CVD

**Fig 3:**
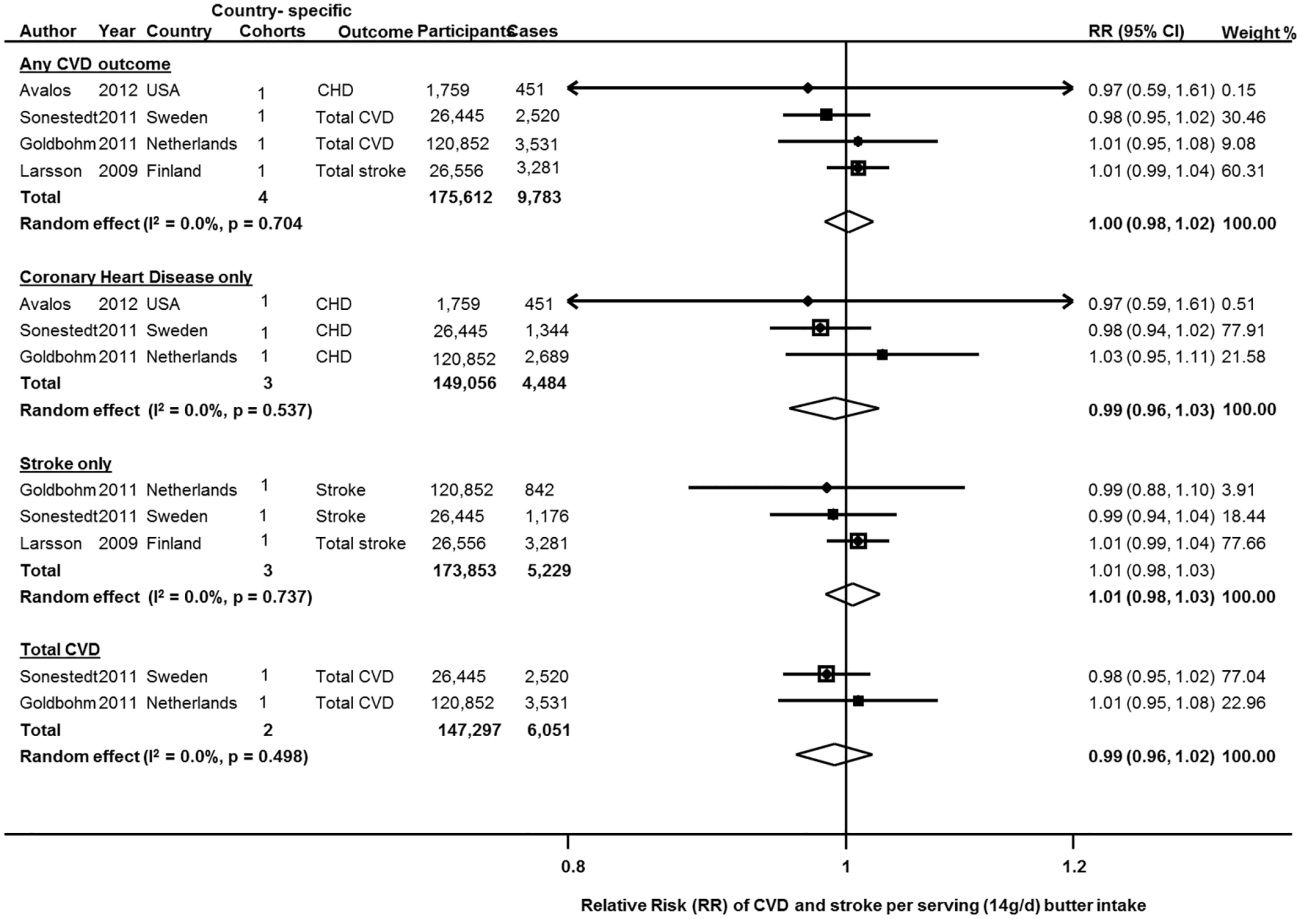
Butter consumption and risk of any and total cardiovascular disease, stroke only and CHD only. Data from 4 prospective cohorts with 175,612 participants and 9,783 cases for CVD, 3 cohorts of 173,853 participants and 5,299 events for stroke, and 3 studies of 149,056 participants and 4,484 cases of CHD. Within-study dose-response RRs were derived from reported linear effects or generalized least-squares trend estimation for studies reporting categories of intake, a pooled using both inverse-variance weighted random and fixed effects meta-analysis. CHD: Coronary Heart Disease; CVD: Cardiovascular Disease; D+L: DerSimonian and Laird random effects; I-V: Inverse-variance fixed effects; RR (95%CI): Relative Risk and 95% Confidence Interval.

Butter consumption and incident CVD (total CVD, CHD, or stroke) was investigated in 5 studies from 4 cohorts, including 175,612 participants and 9,783 cases of any CVD. When pooled, butter intake was not significantly associated with CVD (RR = 1.00 (95%CI = 0.98, 1.02; P = 0.704) (Fig 3). Results were similar with fixed effects; with minimal heterogeneity between studies (I^2^ = 0.0%).

Stroke alone was investigated in 3 studies including 5,299 incident cases (Fig 3); and CHD alone, in 3 studies including 4,484 cases. No significant associations were seen: RR = 1.01 (95%CI = 0.98, 1.03; P = 0.737), and RR = 0.99 (95%CI = 0.96, 1.03; P = 0.537), respectively, without evidence for heterogeneity (I^2^ = 0.0% each).

Total CVD, combining outcomes of CHD and stroke was reported in two cohorts, consisting of 123,497 participants, with 6051 total CVD events (4033 CHD and 2018 stroke). Results from random and fixed effects meta-analysis were identical (RR = 0.99 (95%CI = 0.96, 1.02); P = 0.498). No heterogeneity between the two studies was detected (I^2^ = 0.0%, P = 0.498).

### Type 2 diabetes

**Fig 4:**
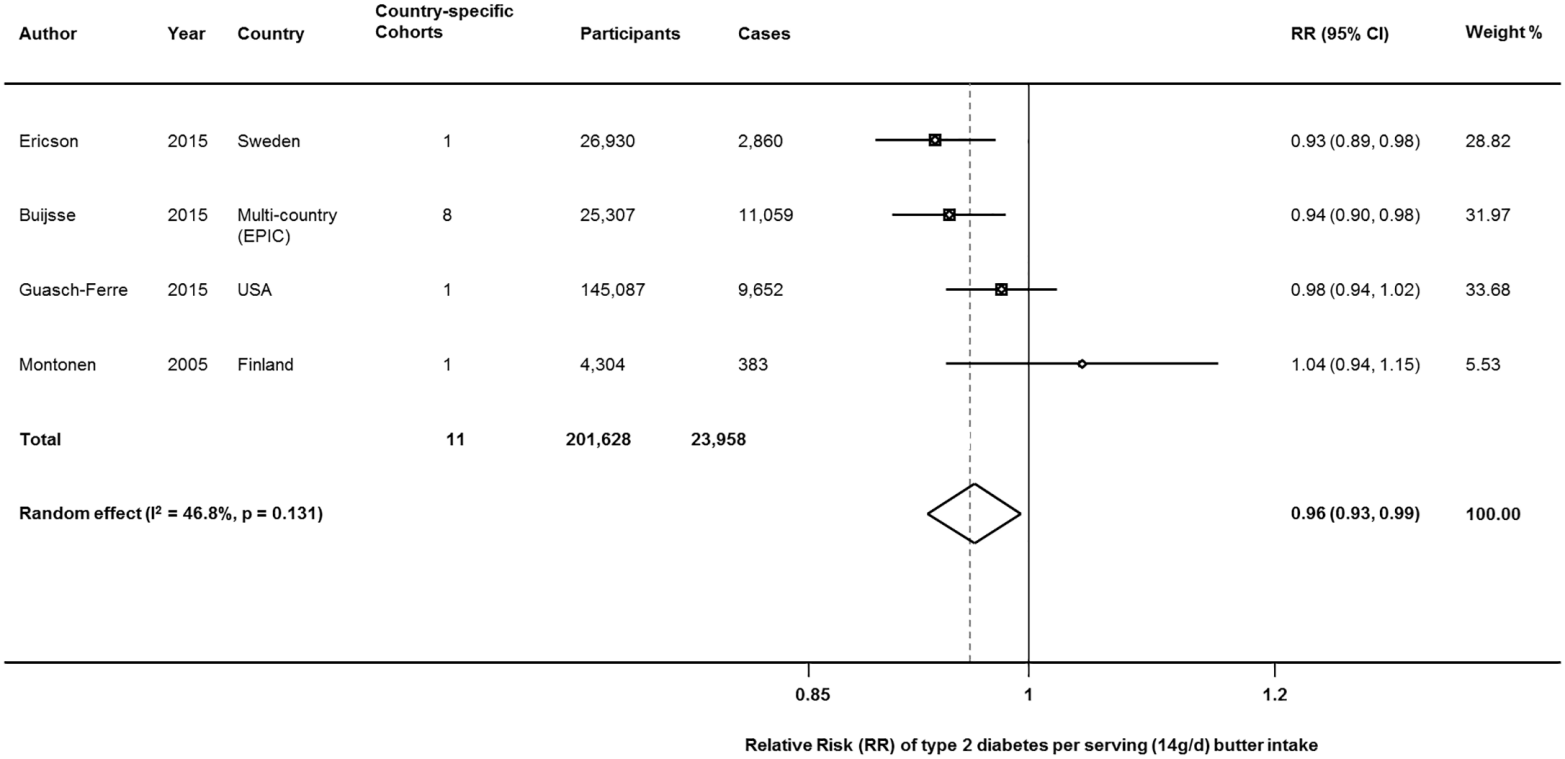
Butter consumption and risk of type 2 diabetes. Within-study dose-response RRs were derived from reported linear effects or generalized least-squares trend estimation for studies reporting categories of intake, a pooled using both inverse-variance weighted random and fixed effects meta-analysis. **D+L**: DerSimonian and Laird random effects; **EPIC**: European Prospective Investigation into Cancer and Nutrition; **I-V**: Inverse-variance fixed effects; **RR (95%CI)**: Relative Risk and 95% Confidence Interval.

Four studies including 11 country-specific cohorts reported on butter consumption and onset of type 2 diabetes [14, 15, 26, 29], including 201,628 participants and 23,954 incident cases. In both random-effects and fixed effects meta-analysis, butter consumption was associated with lower incidence of type 2 diabetes, with 4% lower risk per daily 14g serving: RR = 0.96 (95%CI = 0.93,0.99); P = 0.021). Moderate heterogeneity was seen (I^2^ = 42.1%, p-heterogeneity = 0.131) (Fig 4).

### Subgroup and sensitivity analyses

While total numbers of subjects and cases were large, the relatively low number of separate studies precluded meaningful subgroup analyses by study or participant characteristics, which were therefore not performed. Similarly, potential nonlinearity in dose-response could not be meaningfully evaluated for total mortality. Evidence for nonlinearity was not identified for butter intake and CVD or diabetes (by cubic spline regression, P for nonlinearity = 0.364 and 0.160, respectively).

### Publication bias and small-study effects

Visual inspection of funnel plots and Egger’s test suggested little evidence for asymmetry or presence of small-study effects for any CVD (p = 0.866), stroke (p = 0.913), CHD (p = 0.769), or diabetes (p = 0.369), although the relatively small number of studies limited statistical power of Egger’s test (S1 Fig). Egger’s test could not estimate small-study effects for all-cause mortality (N = 2 studies). No trimming was identified for all-cause mortality or CVD using Duval and Tweedie’s “Trim and Fill” method (S2 Fig). For diabetes, this approach did estimate one missing study, the addition of which resulted in a theoretical corrected pooled estimate of RR = 0.95 (95%CI = 0.93, 0.98; P = 0.001).

## Discussion

In this systematic review and meta-analysis of prospective studies, we found a small positive association between butter consumption and all-cause mortality, no significant association with incident CVD or CVD subtypes, and a modest inverse association with type 2 diabetesNo RCTs of butter intake were identified in our literature search. Because several of the identified reports included multiple country-specific cohorts, the total numbers of nation-specific cohorts, participants, and clinical events appear reasonably robust. Indeed, together these studies included more than 28,000 total deaths, nearly 10,000 cases of incident CVD, and nearly 24,000 cases of incident diabetes. We found limited formal evidence for between-study heterogeneity or publication bias, and all reports had high quality scores. Together, these findings suggest relatively small or neutral associations of butter consumption with long-term health.

Current dietary recommendations on butter and dairy fat are largely based upon predicted effects of specific individual nutrients (e.g., total saturated fat, calcium), rather than actual observed health effects. Our findings add to a growing body of evidence on long-term health effects of specific foods and types of fats. [12, 32, 33] Conventional guidelines on dietary fats have not accounted for their diverse food sources nor the specific individual fatty acid profiles in such foods. [4] Different foods represent complex matrices of nutrients, processing, and food structure, which together influence net health effects. [3, 34] Thus, studying intakes of foods, as in the present investigation, is crucial to elucidate health impact. Our novel results, together with other prior research described below, indicate a need for further funding, evaluation, and reporting on health effects of butter and dairy fat on mechanistic pathways and long-term health outcomes.

While prior meta-analyses have evaluated total dairy or some dairy subtypes and incident diabetes, to our knowledge none have evaluated butter and type 2 diabetes. [6, 12] A meta-analysis of butter and all-cause mortality identified no significant association (highest category vs. lowest: RR = 0.96; 95%CI = 0.95, 1.08) [10], but did not include the more recent large report from Sluik *et al*. [30](258,911 participants, 12,135 deaths) and also included two smaller studies not meeting our inclusion criteria: one having only crude (unadjusted) estimates, [35] and another evaluating polyunsaturated fats or margarine in comparison to butter, rather than butter separately. [36] A meta-analysis evaluating dairy consumption and CVD found no association between butter consumption and stroke (2 cohorts: RR = 0.94; 95%CI = 0.84, 1.06) or CHD (3 cohorts: RR = 1.02, 95%CI = 0.88, 1.20), but only evaluated high vs. low categories of intake rather than conducting dose-response analyses utilizing all available data. [5] Another meta-analysis included dose-response findings on butter consumption and stroke, but not CHD, CVD, diabetes, or all-cause mortality, and arrived at similar findings for stroke as seen in the present study. [11] In comparison to these prior reports, we evaluated up-to-date reports and full dose-response analyses for all-cause mortality, CVD including CHD and stroke, and type 2 diabetes; providing the most comprehensive investigation to-date of butter consumption and risk of long-term major health endpoints.

Our investigation also adds to and expands upon prior studies evaluating other dairy foods and dairy fat biomarkers in relation to cardiometabolic outcomes. In a multi-ethnic US population, serum levels of pentadecanoic acid (15:0), the odd-chain saturated fat most strongly associated with self-reported butter intakes (r = 0.13), were associated with lower CVD and CHD risk. [37] This is consistent with a meta-analysis of odd-chain saturated fat biomarkers demonstrating inverse associations with CHD [33]. A prior meta-analysis of dairy consumption and CVD suggested protective associations with total CVD (for highest vs lowest category of intake: 12% lower risk) and stroke (13% lower risk), with conflicting results for major subtypes of dairy. [5] Dairy fat has also been linked to lower risk of diabetes, based on studies of circulating fatty acid biomarkers [8, 9] and studies of self-reported consumption of dairy products, which have seen protective associations for yogurt and perhaps cheese, and null associations for both low-fat and whole-fat milk. [12, 27]

Given adverse effects of certain dairy fats (e.g. 16:0) on cardiometabolic risk factors such as LDL-cholesterol and fasting glucose [38, 39], our findings suggest potential presence of other mechanistic benefits of butter that might at least partly offset these harms. For instance, saturated fats also increase HDL-C, lower VLDL-C and chylomicron remnants, and lower lipoprotein(a) [40, 41]; while potential cardiometabolic benefits have been identified for calcium, fat-soluble vitamin D, medium-chain saturated fats, branched-chain fats, trace ruminant trans fats, or other processes related to fermentation (e.g. cheese) or active bacterial cultures (e.g. in yogurt). For example, dietary calcium may decrease fatty acid synthase and increase lipolytic activity in adipocytes, [42] reduce blood pressure by modulation of smooth muscle reactivity, [43, 44] and reduce weight gain. [45] Vitamin D may reduce dyslipidemia and improve blood pressure through maintenance of calcium homeostasis, stimulation of insulin production and release, and regulation of the renin-angiotensin-aldosterone system. Higher dairy fat consumption has been linked to lower liver fat and greater hepatic and systemic insulin sensitivity [46] which could relate to inhibition of hepatic de novo lipogenesis by specific dairy fatty acids. [8] Branched-chain fatty acids in dairy fat may promote healthier bacterial microbiome composition and function. Dairy fat also contains monounsaturated fats which might improve glycemic responses and insulin sensitivity. [47, 48] Other dairy-related factors, such as probiotic bacteria in yogurt and menaquinones in fermented milk and cheeses, may improve insulin sensitivity, reduce weight gain, and reduce inflammation through microbiome and vitamin-K related pathways; [49, 50] such pathways would be less relevant for butter, which has been linked to greater weight gain. [51, 52] Clearly, additional mechanistic studies on health effects of butter, dairy fat, and dairy foods are warranted.

Our results suggest relatively small or neutral overall associations of butter with mortality, CVD, and diabetes. These findings should be considered against clear harmful effects of refined grains, starches, and sugars on CVD and diabetes; [53–55] and corresponding benefits of fruits, nuts, legumes, n-6 rich vegetable oils, and possibly other foods such as fish on these endpoints. In sum, these results suggest that health effects of butter should be considered against the alternative choice. For instance, butter may be a more healthful choice than the white bread or potato on which it is commonly spread. In contrast, margarines, spreads, and cooking oils rich in healthful oils, such as soybean, canola, flaxseed, and extra-virgin olive oil, appear to be healthier choices than either butter or refined grains, starches, and sugars. [15, 56, 57] In Guasch-Ferre’s analysis of the Nurses Health Study, substitution of 8 g olive oil for an equivalent amount of butter was associated with an 8% reduction in the risk of type 2 diabetes (RR = 0.92 (95%CI = 0.87, 0.97). [15] Thus, even with an absence of major health associations in the present investigation, healthier (and less healthy) alternatives may be available. Our findings suggest a major focus on eating more or less butter, by itself, may not be linked to large differences in mortality, cardiovascular disease, or diabetes. In sum, our findings do not support a need for major emphasis in dietary guidelines on butter consumption, in comparison to other better established dietary priorities. In any meta-analysis, the effects of potential publication bias should be considered. Such bias increases the probability that large, positive associations, rather than small or null findings, will be published. In this case, the identified studies each reported generally modest or null findings. Considering the number of large prospective studies globally having data on dietary habits (including butter consumption) and these outcomes, it is evident that many additional cohort studies have collected such data but not analyzed or reported their findings. Such “missing,” unpublished studies may be more likely to have null effects. This may be particularly relevant for total mortality, with only 2 identified publications: additional publications might plausibly move findings toward the null. For diabetes, where a larger count of publications allowed better assessment for bias, the “trim and fill” method identified one theoretical missing study, with a protective point estimate.

Our investigation has several strengths. We followed stringent eligibility criteria that maximized inclusion of high quality, comparable studies. Our comprehensive literature search of multiple databases together with author contacts for clarification and missing data maximized statistical power and minimized the possibility of missed reports. While relatively few publications reported on certain outcomes, the identified studies were large, included multiple nation-specific cohorts and thousands of cases, and were of high quality; and as described above, it would be unlikely that publication bias would explain small or null (as opposed to large) associations. The inclusion of generally healthy participants followed since the 1980s and 1990s to the present provided populations generally free of lipid-lowering medications, which might otherwise mask full effects of butter on CVD. The identified cohorts provided a wide range of butter intakes, increasing power to detect an effect, if present. The dose-response analyses maximized use of all reported data, increasing precision.

Potential limitations should be considered. The health effect of any food could be modified by a person’s background diet, genetics, or risk factor profile. This is true for any lifestyle, pharmacologic, or other health intervention—effects may be modified by other treatments or underlying characteristics—but this does not lessen the relevance of evaluating the average population effect. We did not observe any obvious differences in associations based on country or region, where background dietary patterns might differ; but the number of identified studies precluded robust investigation of potential sources of heterogeneity. While the majority of studies adjusted for major demographic, clinical, and dietary covariates, residual confounding may be present. Because butter consumption is associated with generally worse diet patterns and lifestyle habits [58, 59], such residual confounding may overestimate potential harms of butter for mortality, and underestimate potential benefits of butter for CVD or diabetes. Error or bias in measurement of dietary intake from self-reports, as well as the long periods between dietary assessment and follow-up in several studies (10 years or more), may attenuate findings. On the other hand, even with such limitations, many other dietary factors in these and other cohorts have identified significant associations with mortality, CVD, and diabetes, so this is unlikely to be the sole explanation for the null findings. We did not identify any randomized clinical trials of our hard endpoints, although such a long-term trial focused on butter alone might be prohibitively expensive and impractical. Our results are based on best available observational findings, and long-term interventional studies were not found, limiting inference on causality.

In conclusion, the available evidence indicates small or neutral associations of butter consumption with all-cause mortality, CVD, and type 2 diabetes.

## Supporting Information

S1 Fig Funnel plots for butter and mortality, cardiovascular disease, stroke and CHD, and type 2 diabetes. (DOCX)

S2 Fig “Trim and fill” Funnel plots for butter and mortality, cardiovascular disease, stroke and CHD, and diabetes. Additional point representing the ‘filled’ study in the diabetes filled funnel plot is denoted by a square surrounding the data point.
(DOCX)

S1 File MOOSE Guidelines for Meta-Analyses and Systematic Reviews of Observational Studies. (DOCX)

S2 File PRISMA Checklist. (DOC)

S3 File Search strategies for literature review. (DOCX)

S4 File Reference list of excluded publications. (DOCX)

S5 File Standardized estimation strategies for missing data. (DOCX)
