# Supplementary material for: Is Butter Back? A Systematic Review and Meta-Analysis of Butter Consumption and Risk of Cardiovascular Disease, Diabetes, and Total Mortality
Source: PLoS One. 2016 Jun 29;11(6):e0158118. doi: 10.1371/journal.pone.0158118 (PMC4927102; doi:10.1371/journal.pone.0158118)
Supplement: S1 Fig — (DOCX) [file pone.0158118.s001.docx]

| **** | **** |
| --- | --- |
| **All-cause Mortality** | **Any CVD** |
| **** | **** |
| **Stroke** | **CHD** |
| **** |  |
| **Type 2 diabetes** |  |
| **S1 Fig Funnel plots for butter and mortality, cardiovascular disease, stroke and CHD, and type 2 diabetes** | |
