# Supplementary material for: Is Butter Back? A Systematic Review and Meta-Analysis of Butter Consumption and Risk of Cardiovascular Disease, Diabetes, and Total Mortality
Source: PLoS One. 2016 Jun 29;11(6):e0158118. doi: 10.1371/journal.pone.0158118 (PMC4927102; doi:10.1371/journal.pone.0158118)
Supplement: S2 Fig — Additional point representing the ‘filled’ study in the diabetes filled funnel plot is denoted by a square surrounding the data point. (DOCX) [file pone.0158118.s002.docx]

|  |  |
| --- | --- |
| **All-cause mortality** | **Any CVD** |
|  |  |
| **Stroke** | **CHD** |
|  |  |
| **Type 2 diabetes** |  |
| **S2 Fig “Trim and fill” Funnel plots for butter and mortality, cardiovascular disease, stroke and CHD, and diabetes.** Additional point representing the ‘filled’ study in the diabetes filled funnel plot is denoted by a square surrounding the data point. | |
