# Supplementary material for: Is Butter Back? A Systematic Review and Meta-Analysis of Butter Consumption and Risk of Cardiovascular Disease, Diabetes, and Total Mortality
Source: PLoS One. 2016 Jun 29;11(6):e0158118. doi: 10.1371/journal.pone.0158118 (PMC4927102; doi:10.1371/journal.pone.0158118)
Supplement: S1 File — (DOCX) [file pone.0158118.s003.docx]

**S1 File Supporting Information -- MOOSE Guidelines for Meta-Analyses and Systematic Reviews of Observational Studies**

|  | ***Reported on page No*** | ***Excerpt*** |
| --- | --- | --- |
| ***Title*** Identify the study as a meta-analysis (or systematic review) | ***1*** | Is Butter Back? A Systematic Review and Meta-Analysis of Butter consumption and risk of Cardiovascular Disease, Diabetes, and Total Mortality |
| ***Abstract*** Use the journal’s structured format | ***2*** | ***Headings: Background, Methods and findings and Discussion*** |
| ***Introduction* Present** |  |  |
| · The clinical problem | 3 | “For example, growing evidence supports potential metabolic benefits of certain dairy products, such as yogurt and possibly cheese, on risk of type 2 diabetes ([8](#_ENREF_8), [9](#_ENREF_9)), which may even relate to benefits of dairy fat.([10-12](#_ENREF_10)) However, the relationship of butter, which is highest in dairy fat, with diabetes remains unclear. The long-term effects of butter consumption on other major endpoints, such as all-cause mortality and CVD, are also not well-established.” |
| · The hypothesis | n/a |  |
| · A statement of objectives that includes the study population, the condition of interest, the  exposure or intervention, and the outcome(s) considered | 4 | To provide the best evidence-base on long-term relationships of butter consumption with major health endpoints, we conducted a systematic review and meta-analysis of prospective observational studies or randomized clinical trials investigating butter consumption and all-cause mortality, CVD including coronary heart disease (CHD) and stroke, and type 2 diabetes in general populations. |
| ***Sources* Describe** |  |  |
| · Qualifications of searchers (eg, librarians and investigators) | n/a |  |
| · Search strategy, including time period included in the synthesis and keywords | 5 | “We performed a systematic search for all prospective cohort studies and randomized clinical trials examining butter consumption and all-cause mortality, CVD including CHD and stroke, or type 2 diabetes. Electronic searches were performed using PubMed (www.ncbi.nlm.nih.gov/pubmed), EMBASE (www.scopus.com), The Cochrane Library (www.cochranelibrary.com), Web of Knowledge (www.webofscience.com*)*, CAB Abstracts and Global Health (www.ovid.com), CINAHL (www.ebscohost.com) and grey literature searches of SIGLE (www.opengrey.eu) and ZETOC (www.zetoc.mimas.ac.uk/) from the earliest indexing year of each database through May 2015, without language or other restrictions. Search terms included butter, margarine, dairy, dairy products, yogurt, cheese, ghee, animal fat, solid fat, cardiovascular diseases, heart disease, stroke, myocardial infarction, heart attack, cerebrovascular disease, cerebrovascular accident, sudden death, diabetes, mortality and deaths; see **S1 - Search strategies for literature review** for a full listing. For all final included articles, we further performed hand-searches of citation lists and a review of the first 20 related references on PubMed for additional eligible reports.  “ |
| · Effort to include all available studies, including contact with authors | 5-7 | “If references were only available in abstract form (e.g. from meeting proceedings or conference presentations), data were extracted if sufficient detail was available; if not, a relevant publication was searched for in PubMed.  “Missing information in any category was obtained by direct author contact or, if necessary, estimated using a standard approach (see **S3 - Standardized estimation strategies for missing data**).” |
| · Databases and registries searched | 5 | “Electronic searches were performed using PubMed (www.ncbi.nlm.nih.gov/pubmed), EMBASE (www.scopus.com), The Cochrane Library (www.cochranelibrary.com), Web of Knowledge (www.webofscience.com*)*, CAB Abstracts and Global Health (www.ovid.com), CINAHL (www.ebscohost.com) and grey literature searches of SIGLE (www.opengrey.eu) and ZETOC (www.zetoc.mimas.ac.uk/) from the earliest indexing year of each database through May 2015, without language or other restrictions.” |
| · Search software used, name and version, including special features used (eg, explosion) | 5 and supl data | Electronic searches were performed using PubMed (www.ncbi.nlm.nih.gov/pubmed), EMBASE (www.scopus.com), The Cochrane Library (www.cochranelibrary.com), Web of Knowledge (www.webofscience.com*)*, CAB Abstracts and Global Health (www.ovid.com), CINAHL (www.ebscohost.com) and grey literature searches of SIGLE (www.opengrey.eu) and ZETOC (www.zetoc.mimas.ac.uk/) from the earliest indexing year of each database through May 2015, without language or other restrictions.  Search terms included butter, margarine, dairy, dairy products, yogurt, cheese, ghee, animal fat, solid fat, cardiovascular diseases, heart disease, stroke, myocardial infarction, heart attack, cerebrovascular disease, cerebrovascular accident, sudden death, diabetes, mortality and deaths; see **S1 - Search strategies for literature review** for a full listing. For all final included articles, we further performed hand-searches of citation lists and a review of the first 20 related references on PubMed for additional eligible reports. |
| · Use of hand searching (eg, reference lists of obtained articles) | 5 | “For all final included articles, we further performed hand-searches of citation lists and a review of the first 20 related references on PubMed for additional eligible reports.” |
| · List of citations located and those excluded, including justification | Table 1 and Supplemental data |  |
| · Method of addressing articles published in languages other than English | n/a | None found |
| · Method of handling abstracts and unpublished studies | 6 | . If references were only available in abstract form (e.g. from meeting proceedings or conference presentations), data were extracted if sufficient detail was available; if not, a relevant publication was searched for in PubMed. |
| · Description of any contact with authors | 7 and acknowledgement | When energy intake was included as a covariate, body mass index was not considered to be an intermediate variable, so models adjusting for body mass index were extracted (this only arose in one study, by Buijsse *et al. (*[*17*](#_ENREF_17)*)*[*)*](#_ENREF_17)*.* The effect results from the Guasch-Ferre *et al.(*[*18*](#_ENREF_18)*)* study were estimated using the models of risk of diabetes associated with substitution of olive oil for equivalent amounts of butter, and our results were confirmed and validated by contact with the authors. |
| ***Study Selection* Describe** |  |  |
| · Types of study designs considered | 6 | We searched for all randomized controlled trials or prospective cohorts (cohort, nested case-subcohort, nested case-control) conducted in adults (18+ y) that provided a multivariate-adjusted effect estimate (or unadjusted effect estimate in trials) and measure of statistical uncertainty of the relationship between total or added butter and all-cause mortality, incident CVD including CHD or stroke, and incident diabetes. |
| · Relevance or appropriateness of studies gathered for assessing the hypothesis to be tested | 6 | We searched for all randomized controlled trials or prospective cohorts (cohort, nested case-subcohort, nested case-control) conducted in adults (18+ y) that provided a multivariate-adjusted effect estimate (or unadjusted effect estimate in trials) and measure of statistical uncertainty of the relationship between total or added butter and all-cause mortality, incident CVD including CHD or stroke, and incident diabetes. |
| · Rationale for the selection and coding of data (eg, sound clinical principles or convenience) | 6-7 | Data from the included studies were independently extracted in duplicate by two investigators using a standardized and piloted electronic form (Microsoft Excel). Any differences in extraction were resolved by consensus. Information was extracted on the publication (first author name, contact information, publication year), study details (name, location, design), population (age, gender, race, socioeconomic status, body mass index), sample size, dates of recruitment, duration of follow-up, dietary assessment (dates, method, definition, categories), outcome(s) (assessment method, definition), covariates and analysis methods, and multivariate-adjusted effect estimates and associated uncertainty. To evaluate dose-response, we extracted continuous effect estimates when available; and for categorical analyses, collected additional information on median exposure, number of participants or person-years, and number of events in each category. Missing information in any category was obtained by direct author contact or, if necessary, estimated using a standard approach (see **S3 - Standardized estimation strategies for missing data**). |
| · Documentation of how data were classified and coded (eg, multiple raters, blinding, and  interrater reliability) | 6-7 | Data from the included studies were independently extracted in duplicate by two investigators using a standardized and piloted electronic form (Microsoft Excel). Any differences in extraction were resolved by consensus. Information was extracted on the publication (first author name, contact information, publication year), study details (name, location, design), population (age, gender, race, socioeconomic status, body mass index), sample size, dates of recruitment, duration of follow-up, dietary assessment (dates, method, definition, categories), outcome(s) (assessment method, definition), covariates and analysis methods, and multivariate-adjusted effect estimates and associated uncertainty. To evaluate dose-response, we extracted continuous effect estimates when available; and for categorical analyses, collected additional information on median exposure, number of participants or person-years, and number of events in each category. Missing information in any category was obtained by direct author contact or, if necessary, estimated using a standard approach (see **S3 - Standardized estimation strategies for missing data**). |
| · Assessment of confounding (eg, comparability of cases and controls in studies where  appropriate) | 7 | If the main multivariable model included covariates which could either be confounders or intermediates, this was utilized rather than a model with crude or minimal covariate adjustment |
| · Assessment of study quality, including blinding of quality assessors; stratification or  regression on possible predictors of study results | 7-8 | We adapted the Newcastle-Ottawa quality scale ([19](#_ENREF_19)) to assess study quality, based on five criteria evaluating the reporting and appropriateness/representativeness of participant inclusion and exclusion criteria, participant attrition, control for confounding, assessment of exposure, and assessment of outcome. One point was allocated per criterion met, the sum of which provided an overall quality score. A score between 0 and 3 was considered lower-quality; and 4 to 5, higher-quality. Quality scores were assessed independently and in duplicate by two investigators, with any differences resolved by consensus. |
| · Assessment of heterogeneity | 8 | Heterogeneity between studies was quantified using the I^2^ statistic, with statistical significance evaluated by the Q statistic. ([24](#_ENREF_24)) We considered I^2^ values of 25% and 75% as thresholds for low, moderate, and high heterogeneity, respectively. We planned pre-specified subgroup analyses to further explore potential heterogeneity in results by gender, population mean age and body mass index, duration of follow-up, and study quality score. Restricted cubic spline models^(^[^25^](#_ENREF_25)^)^ with knots at the 25th, 50th, and 75th percentiles were used to examine potential nonlinear relations. |
| · Statistical methods (eg, complete description of fixed or random effects models, justification  of whether the chosen models account for predictors of study results, dose-response models,  or cumulative meta-analysis) in sufficient detail to be replicated | 8 | Reported hazard ratios were assumed to approximate relative risks (RRs). We used the two-stage generalized least-squares trend estimation method described by Greenland and Longnecker ([20](#_ENREF_20), [21](#_ENREF_21)) to perform dose-response analysis and compute study-specific linear estimates and 95% CIs across categories of butter intake. Butter intakes across studies were standardized at the study level to 14 g/d, corresponding to one United Stated Department of Agriculture-defined serving.([22](#_ENREF_22)) Study-specific dose-response estimates were then pooled to derive an overall estimate using inverse-variance weighted DerSimonian and Laird meta-analysis with random effects.([23](#_ENREF_23)) Because random effects can result in larger weights for small outlier studies, we also conducted fixed effects meta-analysis for comparison. For reports presenting results only by study subgroups (e.g., men, women), we first pooled the study-specific subgroups using fixed-effect meta-analysis to obtain a single estimate per study. |
|  |  |  |
| ***Results* Present** |  |  |
| · A graph summarizing individual study estimates and the overall estimate | Fig 1-3 |  |
| · A table giving descriptive information for each included study | Table 1 |  |
| · Results of sensitivity testing (eg, subgroup analysis) | 10-11 | While total numbers of subjects and cases were large, the relatively low number of separate studies precluded meaningful subgroup analyses by study or participant characteristics, which were therefore not performed. Similarly, potential nonlinearity in dose-response could not be meaningfully evaluated for total mortality. Evidence for nonlinearity was not identified for butter intake and CVD or diabetes (by cubic spline regression, P for nonlinearity=0.364 and 0.160, respectively). |
| · Indication of statistical uncertainty of findings | 9-11 | Cf 95%CI and P-values reported for all findings |
| ***Discussion* Discuss** |  |  |
| · Strengths and weaknesses | 20 | Our investigation has several strengths. We followed stringent eligibility criteria that maximized inclusion of higher quality, comparable studies. Our comprehensive literature search of multiple databases together with author contacts for clarification and missing data maximized statistical power and minimized the possibility of missed reports. The inclusion of generally healthy participants followed since the 1980s and 1990s to the present provided populations generally free of lipid-lowering medications, which could mask full effects of butter on CVD. The identified cohorts provided a wide range of butter intakes, increasing power to detect an effect, if present. The dose-response analyses maximized use of all reported data, increasing precision.  Potential limitations should be considered. We identified relatively few individual studies reporting on some outcomes, in particular all-cause mortality. Yet, identified studies were large, included thousands of cases, and were of higher quality; and it seems unlikely that publication bias could explain null or protective findings for CVD and diabetes. The number of studies precluded meaningful investigation of potential sources of heterogeneity and publication bias. Residual confounding may be present in cohort studies; because butter consumption is associated with generally worse diets and lifestyle([62](#_ENREF_62), [63](#_ENREF_63)), this may lead to upward bias and overestimation of risk (or underestimation of benefit). On the other hand, the majority of studies adjusted for major demographic, clinical, and dietary covariates. We did not identify any randomized clinical trials of our hard endpoints, although such a long-term trial focused on butter alone might be prohibitively expensive and impractical. |
| · Potential biases in the review process (eg, publication bias) | 12 | Given the number of studies reporting on each outcome, the potential effects of unidentified publication bias should be carefully considered. Publication bias produces higher probability that large, positive associations will be published, rather than null or unexpected findings. In this case, the published studies each generally reported null findings or, for diabetes, results opposite to conventionally expected associations. Considering the number of large prospective studies globally having data on dietary habits (including butter consumption) and these outcomes, it is evident that many additional cohort studies have collected such data but not analyzed or reported their findings. Such missing studies might be more likely to have null or unexpected protective effects. This may be particularly relevant for total mortality, only reported on by 2 studies. For diabetes, where a larger count of studies allowed better assessment for publication bias, the “trim and fill” method identified one theoretical missing study with a protective point estimate.  Error or bias in measurement of dietary intake from self-reports, as well as the long periods between dietary assessment and follow-up in several studies (10 years or more) which would furthermore attenuate findings towards the null. On the other hand, even with such limitations, many other dietary factors in these and other cohorts have significant associations with mortality, CVD, and diabetes, so this is unlikely to be the sole explanation for the null findings. |
| · Justification for exclusion (eg, exclusion of non–English-language citations) | 13 | While prior meta-analyses have evaluated total dairy or some dairy subtypes and incident diabetes([9](#_ENREF_9), [15](#_ENREF_15)), to our knowledge there have been no prior meta-analyses of butter and type 2 diabetes. A prior meta-analysis of butter and all-cause mortality identified no significant association (highest category vs. lowest: RR=0.96; 95%CI=0.95, 1.08)([13](#_ENREF_13)), but did not include the more recent large report from Sluik *et al*.([33](#_ENREF_33)) (258,911 participants, 12,135 deaths) while also including two smaller studies not actually meeting our criteria for inclusion: one providing only crude (unadjusted) estimates,([60](#_ENREF_60)) and another evaluating polyunsaturated fats or margarine in comparison to butter, rather than butter separately.([61](#_ENREF_61)) Another meta-analysis evaluating dairy consumption and CVD ([8](#_ENREF_8)) found no association between butter consumption and stroke (2 cohorts: RR=0.94; 95%CI=0.84, 1.06) or CHD (3 cohorts: RR=1.02, 95%CI=0.88, 1.20), but only evaluated high vs. low categories of intake rather than conducting dose-response analyses utilizing all available data. Another meta-analysis included dose-response findings on butter consumption and stroke, but not CHD, CVD, diabetes, or all-cause mortality, and arrived at similar findings for stroke as seen in the present study.([14](#_ENREF_14)) In comparison to these prior reports, we evaluated up-to-date reports and full dose-response analyses for all-cause mortality, CVD including CHD and stroke, and type 2 diabetes; providing the most comprehensive look at butter consumption and risk of long-term major health endpoints. |
| · Assessment of quality of included studies | 15 | Given the large numbers of events in the identified studies, together with the conventional direction of publication bias, one would expect that inclusion of such additional unpublished studies might reverse the small risk seen for total mortality, confirm null associations for CVD, and strengthen (or move to the null) possible protective effects for diabetes, rather than resulting in any new strong adverse effects of butter. |
| · Consideration of alternative explanations for observed results | 12-14 | Together, these findings suggest mixed evidence for long-term health effects of butter consumption. Given the number of studies reporting on each outcome, the potential effects of unidentified publication bias should be carefully considered. Publication bias produces higher probability that large, positive associations will be published, rather than null or unexpected findings. In this case, the published studies each generally reported null findings or, for diabetes, results opposite to conventionally expected associations. Considering the number of large prospective studies globally having data on dietary habits (including butter consumption) and these outcomes, it is evident that many additional cohort studies have collected such data but not analyzed or reported their findings. Such missing studies might be more likely to have null or unexpected protective effects. This may be particularly relevant for total mortality, only reported on by 2 studies. For diabetes, where a larger count of studies allowed better assessment for publication bias, the “trim and fill” method identified one theoretical missing study with a protective point estimate. |
| · Generalization of the conclusions (ie, appropriate for the data presented and within the domain | 16 | Our findings add to a growing body of evidence on potential health effects of dietary fats, and dairy fat in particular, on mortality and cardiometabolic health.([3](#_ENREF_3), [15](#_ENREF_15), [35](#_ENREF_35)) Recommendations on dietary fats traditionally have not accounted for their diverse food sources or specific individual fatty acid profiles in such foods.([7](#_ENREF_7)) Different foods represent complex matrices of nutrients, processing, and food structure, which together influence net health effects.([6](#_ENREF_6), [36](#_ENREF_36)) The health effect of a particular food could also be modified by one’s overall diet pattern, and that findings from observational studies or randomized trials evaluating a single food should be evaluated in that context. Nonetheless, the same is true for any lifestyle, pharmacologic or other health intervention – effects may be modified by other treatments or underlying characteristics – which does not lessen the relevance of evaluating the average population effect. |
| of the literature review) |  |  |
| · Guidelines for future research | 15-16 | Publication bias produces higher probability that large, positive associations will be published, rather than null or unexpected findings. In this case, the published studies each generally reported null findings or, for diabetes, results opposite to conventionally expected associations. Considering the number of large prospective studies globally having data on dietary habits (including butter consumption) and these outcomes, it is evident that many additional cohort studies have collected such data but not analyzed or reported their findings. Such missing studies might be more likely to have null or unexpected protective effects. This may be particularly relevant for total mortality, only reported on by 2 studies |
| · Disclosure of funding source | n/a | Funding disclorure in PLOS application not manuscrcipt. |

*Modified from Stroup DF, Berlin JA, Morton SC, Olkin I, Williamson GD, Rennie D, et al. Meta-analysis of observational

studies in epidemiology: a proposal for reporting. Meta-analysis Of Observational Studies in Epidemiology (MOOSE) group.

JAMA 2000;283:2008–12. Copyrighted © 2000, American Medical Association. All rights reserved.
