# Supplementary material for: Is Butter Back? A Systematic Review and Meta-Analysis of Butter Consumption and Risk of Cardiovascular Disease, Diabetes, and Total Mortality
Source: PLoS One. 2016 Jun 29;11(6):e0158118. doi: 10.1371/journal.pone.0158118 (PMC4927102; doi:10.1371/journal.pone.0158118)
Supplement: S3 File — (DOCX) [file pone.0158118.s005.docx]

**S3 File. Supporting Information. Search strategies for literature review**

**Database: The Cochrane Library**

**Date:** 11 May 2015

**Search:**

1. ((MeSH descriptor: [Dairy Products] explode all trees) OR (dairy or butter or margarine or yogurt or yoghurt or cheese or ghee or "animal fat" or "animal fats" or "solid fat" or "solid fats":ti,ab,kw))(Word variations have been searched)
2. "cardiovascular disease" or "cardiovascular diseases" or "heart disease" or "heart diseases" or stroke* or "myocardial infarction" or "myocardial infarctions" or "heart attack" or "heart attacks" or "cerebrovascular disease" or "cerebrovascular diseases" or "cerebral infarction" or "cerebral infarctions" or "cerebrovascular accident" or "cerebrovascular accidents" or "sudden death" or "sudden deaths" or diabetes or mortality or death:ti,ab,kw (Word variations have been searched)
3. 1 AND 2

**Records**: N= 432

**Database: Medline**

**Date:** 7 May 2015

**Search**:

1. (dairy[tiab] OR dairy products[mesh:noexp] OR butter[MeSH Terms] OR butter[Title/Abstract] OR butters[Title/Abstract] OR margarine[MeSH Terms] OR margarine[Title/Abstract] OR yoghurt[MeSH Terms] OR yoghurt[Title/Abstract] OR yogurt[Title/Abstract] OR cheese[MeSH Terms] OR cheese[Title/Abstract] OR ghee[Title/Abstract] OR ghees[Title/Abstract] OR “animal fat”[Title/Abstract] OR “animal fats”[Title/Abstract] OR “solid fat”[Title/Abstract] OR “solid fats”[Title/Abstract])
2. (cardiovascular diseases[MeSH Terms] OR “cardiovascular disease”[Title/Abstract] OR “cardiovascular diseases”[Title/Abstract] OR “heart disease”[Title/Abstract] OR “heart diseases”[Title/Abstract] OR stroke*[Title/Abstract] OR “myocardial infarction”[Title/Abstract] OR “myocardial infarctions”[Title/Abstract] OR “heart attack”[Title/Abstract] OR “heart attacks”[Title/Abstract] OR “cerebrovascular disease”[Title/Abstract] OR “cerebrovascular diseases”[Title/Abstract] OR “cerebral infarction”[Title/Abstract] OR “cerebral infarctions”[Title/Abstract] OR “cerebrovascular accident”[Title/Abstract] OR “cerebrovascular accidents”[Title/Abstract] OR “sudden death”[Title/Abstract] OR “sudden deaths”[Title/Abstract] OR diabetes[MeSH Terms] OR diabetes[Title/Abstract] OR mortality[MeSH Terms] OR mortality[Title/Abstract] OR death[MeSH Terms] OR death*[Title/Abstract]) (No filter)
3. 1 AND 2

**Records: 3997**

**Database: Embase via SCOPUS**

**Date**: 11 May 2015

**Search:**

1. TITLE-ABS-KEY (dairy OR "dairy products" OR butter OR margarine* OR yoghurt* OR yogurt* OR cheese* OR ghee* OR "animal fat" OR "animal fats" OR "solid fat" OR "solid fats"))
2. (TITLE-ABS-KEY("cardiovascular disease" OR "cardiovascular diseases" OR "heart disease" OR "heart diseases" OR stroke* OR "myocardial infarction" OR "myocardial infarctions" OR "heart attack" OR "heart attacks" OR "cerebrovascular disease" OR "cerebrovascular diseases" OR "cerebral infarction" OR "cerebral infarctions “OR "cerebrovascular accident" OR "cerebrovascular accidents" OR "sudden death" OR "sudden deaths" OR diabetes OR mortality OR death))
3. (EXCLUDE(DOCTYPE, "ar") OR EXCLUDE(DOCTYPE, "re") OR EXCLUDE(DOCTYPE, "cp"))
4. (EXCLUDE(EXACTKEYWORD, "Animals") OR EXCLUDE(EXACTKEYWORD, "Animal") OR EXCLUDE (EXACTKEYWORD, "Cattle") OR EXCLUDE(EXACTKEYWORD, "Cattle Diseases") OR EXCLUDE (EXACTKEYWORD, "Cattle disease") OR EXCLUDE(EXACTKEYWORD, "Animal disease"))
5. 1 AND 2 AND 3 AND 4

**Records**: =476

***Web of Knowledge***

**Date**:

**Search**:

1. TS=("cardiovascular disease" or "cardiovascular diseases" or "heart disease" or "heart diseases" or stroke* or "myocardial infarction" or "myocardial infarctions" or "heart attack" or "heart attacks" or "cerebrovascular disease" or "cerebrovascular diseases" or "cerebral infarction" or "cerebral infarctions" or "cerebrovascular accident" or "cerebrovascular accidents" or "sudden death" or "sudden deaths" or diabetes or mortality or death or death*) Indexes=SCI-EXPANDED, CPCI-S Timespan=All years
2. TS=(dairy or "dairy products" or butter or margarine* or yoghurt* or yogurt* or cheese* or ghee* or "animal fat" or "animal fats" or "solid fat" or "solid fats") Indexes=SCI-EXPANDED, CPCI-S Timespan=All years
3. 1 AND 2 [excluding] DOCUMENT TYPES:( EDITORIAL MATERIAL OR BOOK CHAPTER OR REPRINT OR LETTER OR NEWS ITEM OR CORRECTION ADDITION OR NOTE OR CORRECTION OR DISCUSSION ) AND [excluding] RESEARCH AREAS:( VETERINARY SCIENCES ) AND [excluding] WEB OF SCIENCE CATEGORIES:( AGRICULTURE DAIRY ANIMAL SCIENCE ) Indexes=SCI-EXPANDED, CPCI-S Timespan=All years

**Records**: 3547

**Database:CAB abstracts and Global Health**

**Date:** 11 May 2015

**Search:**

1. (dairy or "dairy products" or butter or margarine* or yoghurt* or yogurt* or cheese* or ghee* or "animal fat" or "animal fats" or "solid fat" or "solid fats").ab,sh,ti.
2. ("cardiovascular disease" or "cardiovascular diseases" or "heart disease" or "heart diseases" or stroke* or "myocardial infarction" or "myocardial infarctions" or "heart attack" or "heart attacks" or "cerebrovascular disease" or "cerebrovascular diseases" or "cerebral infarction" or "cerebral infarctions" or "cerebrovascular accident" or "cerebrovascular accidents" or "sudden death" or "sudden deaths" or diabetes or mortality or death or death*).ab,sh,ti.
3. Human.hw.
4. 1 AND 2 AND 3

**Records**: 2134

**Database: CINAHL via EBSCO**

**Date:** 11 May 2015

**Search:**

1. "TI ( (dairy OR dairy products OR butter OR butter OR butters OR margarine OR margarine OR yoghurt OR yoghurt OR yogurt OR cheese OR cheese OR ghee OR ghees OR “animal fat” OR “animal fats” OR “solid fat” OR “solid fats”) OR MW ( (dairy OR dairy products OR butter OR butter OR butters OR margarine OR margarine OR yoghurt OR yoghurt OR yogurt OR cheese OR cheese OR ghee OR ghees OR “animal fat” OR “animal fats” OR “solid fat” OR “solid fats”) OR AB ( (dairy OR dairy products OR butter OR butter OR butters OR margarine OR margarine OR yoghurt OR yoghurt OR yogurt OR cheese OR cheese OR ghee OR ghees OR “animal fat” OR “animal fats” OR “solid fat” OR “solid fats”)
2. TI( (cardiovascular diseases OR “cardiovascular disease” OR “cardiovascular diseases” OR “heart disease” OR “heart diseases” OR stroke* OR “myocardial infarction” OR “myocardial infarctions” OR “heart attack” OR “heart attacks” OR “cerebrovascular disease” OR “cerebrovascular diseases” OR “cerebral infarction” OR “cerebral infarctions” OR “cerebrovascular accident” OR “cerebrovascular accidents” OR “sudden death” OR “sudden deaths” OR diabetes OR diabetes OR mortality OR mortality OR death OR death*) ) OR MW ( (dairy OR dairy products OR butter OR butter OR butters OR margarine OR margarine OR yoghurt OR yoghurt OR yogurt OR cheese OR cheese OR ghee OR ghees OR “animal fat” OR “animal fats” OR “solid fat” OR “solid fats”) OR MW (cardiovascular diseases OR “cardiovascular disease” OR “cardiovascular diseases” OR “heart disease” OR “heart diseases” OR stroke* OR “myocardial infarction” OR “myocardial infarctions” OR “heart attack” OR “heart attacks” OR “cerebrovascular disease” OR “cerebrovascular diseases” OR “cerebral infarction” OR “cerebral infarctions” OR “cerebrovascular accident” OR “cerebrovascular accidents” OR “sudden death” OR “sudden deaths” OR diabetes OR diabetes OR mortality OR mortality OR death OR death*) ) OR AB (cardiovascular diseases OR “cardiovascular disease” OR “cardiovascular diseases” OR “heart disease” OR “heart diseases” OR stroke* OR “myocardial infarction” OR “myocardial infarctions” OR “heart attack” OR “heart attacks” OR “cerebrovascular disease” OR “cerebrovascular diseases” OR “cerebral infarction” OR “cerebral infarctions” OR “cerebrovascular accident” OR “cerebrovascular accidents” OR “sudden death” OR “sudden deaths” OR diabetes OR diabetes OR mortality OR mortality OR death OR death*) )
3. 1 AND 2

**Records**: 537

**Database: ZETOC**

**Date:** 11 May 2015

**Search**: “butter” (no OR Boolean in ZETOC so broad) in conference field

**Records**:312

**Database: SIGLE**

**Date:** 11 May 2015

**Search:**

1. (dairy OR “dairy products” OR butter* OR margarine* OR yoghurt* OR yogurt* OR cheese* OR ghee* OR “animal fat” OR “animal fats” OR “solid fat” OR “solid fats”)
2. (“cardiovascular disease” OR “cardiovascular diseases” OR “heart disease” OR “heart diseases” OR stroke* OR “myocardial infarction” OR “myocardial infarctions” OR “heart attack” OR “heart attacks” OR “cerebrovascular disease” OR “cerebrovascular diseases” OR “cerebral infarction” OR “cerebral infarctions” OR “cerebrovascular accident” OR “cerebrovascular accidents” OR “sudden death” OR “sudden deaths” OR diabetes OR mortality OR death OR death*)
3. 1 AND 3

**Records: 46**
