# Supplementary material for: Is Butter Back? A Systematic Review and Meta-Analysis of Butter Consumption and Risk of Cardiovascular Disease, Diabetes, and Total Mortality
Source: PLoS One. 2016 Jun 29;11(6):e0158118. doi: 10.1371/journal.pone.0158118 (PMC4927102; doi:10.1371/journal.pone.0158118)
Supplement: S4 File — (DOCX) [file pone.0158118.s006.docx]

**S4 File. Supporting Information. Reference list of excluded publications**

**List of excluded studies with reasons (N=171):**

**Did not report on primary research, was ineligible article type or study design (n=64)**

Aune D, Norat T, Romundstad P, Vatten LJ. Dairy products and the risk of type 2 diabetes: a systematic review and dose-response meta-analysis of cohort studies. The American journal of clinical nutrition. Oct 2013;98(4):1066-1083.

Chen M, Sun Q, Giovannucci E, et al. Dairy consumption and risk of type 2 diabetes: 3 cohorts of US adults and an updated meta-analysis. BMC medicine. 2014;12:215.

Hu D, Huang J, Wang Y, Zhang D, Qu Y. Dairy foods and risk of stroke: a meta-analysis of prospective cohort studies. Nutrition, metabolism, and cardiovascular diseases : NMCD. May 2014;24(5):460-469.

O'Sullivan TA, Hafekost K, Mitrou F, Lawrence D. Food sources of saturated fat and the association with mortality: a meta-analysis. American journal of public health. Sep 2013;103(9):e31-42.

O'Sullivan TA, Hafekost K, Mitrou F, Lawrence D. Food sources of saturated fat and the association with mortality: a meta-analysis. American journal of public health. Sep 2013;103(9):e31-42.

Qin LQ, Xu JY, Han SF, Zhang ZL, Zhao YY, Szeto IM. Dairy consumption and risk of cardiovascular disease: an updated meta-analysis of prospective cohort studies. Asia Pacific journal of clinical nutrition. 2015;24(1):90-100.

Akter S, Kurotani K, Nanri A, et al. Dairy consumption is associated with decreased insulin resistance among the Japanese. Nutr. Res. Apr 2013;33(4):286-292.

Astrup A. Yogurt and dairy product consumption to prevent cardiometabolic diseases: epidemiologic and experimental studies. The American journal of clinical nutrition. May 2014;99(5 Suppl):1235s-1242s.

Bamia C, Orfanos P, Ferrari P, et al. Dietary patterns among older Europeans: the EPIC-Elderly study. British Journal of Nutrition. Jul 2005;94(1):100-113.

Bilenko N, Fraser D, Vardi H, Shai I, Shahar DR. Mediterranean diet and cardiovascular diseases in an Israeli population. Preventive medicine. Mar 2005;40(3):299-305.

Candido FG, Ton WT, Alfenas Rde C. Dairy products consumption versus type 2 diabetes prevention and treatment; a review of recent findings from human studies. Nutricion hospitalaria. Sep-Oct 2013;28(5):1384-1395.

Clifton PM. Protein and coronary heart disease: the role of different protein sources. Current atherosclerosis reports. Dec 2011;13(6):493-498.

Correa-Matos NJ, Vaghefi SB. Dairy Foods and Cardiovascular Diseases. Bioactive Food as Dietary Interventions for Cardiovascular Disease2013:319-332.

de Lorgeril M, Salen P. The Mediterranean-style diet for the prevention of cardiovascular diseases. Public health nutrition. Feb 2006;9(1a):118-123.

Delavar MA, Lye MS, Khor GL, Hassan S, Hanachi P. Dietary patterns and the metabolic syndrome in middle aged women, Babol, Iran. Asia Pacific journal of clinical nutrition. 2009;18(2):285-292.

DiBello JR, Kraft P, McGarvey ST, Goldberg R, Campos H, Baylin A. Comparison of 3 methods for identifying dietary patterns associated with risk of disease. American journal of epidemiology. Dec 15 2008;168(12):1433-1443.

Ellis IH. Communicable disease associated with milk and dairy products. British medical journal (Clinical research ed.). Sep 25 1982;285(6345):895.

Elmstahl S, Holmqvist O, Gullberg B, Johansson U, Berglund G. Dietary patterns in high and low consumers of meat in a Swedish cohort study. Appetite. Apr 1999;32(2):191-206.

Elwood PC, Givens DI, Beswick AD, Fehily AM, Pickering JE, Gallacher J. The survival advantage of milk and dairy consumption: an overview of evidence from cohort studies of vascular diseases, diabetes and cancer. Journal of the American College of Nutrition. Dec 2008;27(6):723s-734s.

Ford DW, Jensen GL, Hartman TJ, Wray L, Smiciklas-Wright H. Association between dietary quality and mortality in older adults: a review of the epidemiological evidence. Journal of nutrition in gerontology and geriatrics. 2013;32(2):85-105.

Fraser G. Commentary: Protection from stroke by eating animal foods? Surely not! International journal of epidemiology. Aug 2003;32(4):543-545.

Gao D, Ning N, Wang C, et al. Dairy products consumption and risk of type 2 diabetes: systematic review and dose-response meta-analysis. PloS one. 2013;8(9):e73965.

Gayet-Boyer C, Tenenhaus-Aziza F, Prunet C, et al. Is there a linear relationship between the dose of ruminant trans-fatty acids and cardiovascular risk markers in healthy subjects: results from a systematic review and meta-regression of randomised clinical trials. British Journal of Nutrition. Dec 2014;112(12):1914-1922.

Gebauer SK, Chardigny JM, Jakobsen MU, et al. Effects of ruminant trans fatty acids on cardiovascular disease and cancer: a comprehensive review of epidemiological, clinical, and mechanistic studies. Advances in nutrition (Bethesda, Md.). Jul 2011;2(4):332-354.

Gibson RA, Makrides M, Smithers LG, Voevodin M, Sinclair AJ. The effect of dairy foods on CHD: a systematic review of prospective cohort studies. The British journal of nutrition. Nov 2009;102(9):1267-1275.

Hirahatake KM, Slavin JL, Maki KC, Adams SH. Associations between dairy foods, diabetes, and metabolic health: potential mechanisms and future directions. Metabolism: clinical and experimental. May 2014;63(5):618-627.

Hu D, Huang J, Wang Y, Zhang D, Qu Y. Dairy foods and risk of stroke: a meta-analysis of prospective cohort studies. Nutrition, metabolism, and cardiovascular diseases : NMCD. May 2014;24(5):460-469.

Huth PJ, Park KM. Influence of dairy product and milk fat consumption on cardiovascular disease risk: a review of the evidence. Advances in nutrition (Bethesda, Md.). May 2012;3(3):266-285.

Ismail J, Jafar TH, Jafary FH, White F, Faruqui AM, Chaturvedi N. Risk factors for non-fatal myocardial infarction in young South Asian adults. Heart (British Cardiac Society). Mar 2004;90(3):259-263.

Jakobsen MU, Bysted A, Andersen NL, et al. Intake of ruminant trans fatty acids and risk of coronary heart disease - An overview. Atheroscler. Suppl. May 2006;7(2):9-11.

Kontogianni MD, Panagiotakos DB, Chrysohoou C, Pitsavos C, Stefanadis C. Modelling dairy intake on the development of acute coronary syndromes: the CARDIO2000 study. European journal of cardiovascular prevention and rehabilitation : official journal of the European Society of Cardiology, Working Groups on Epidemiology & Prevention and Cardiac Rehabilitation and Exercise Physiology. Oct 2006;13(5):791-797.

Kratz M, Baars T, Guyenet S. The relationship between high-fat dairy consumption and obesity, cardiovascular, and metabolic disease. European journal of nutrition. Feb 2013;52(1):1-24.

Kwak HS, Ganesan P, Mijan MA. Butter, Ghee, and Cream Products. Milk and Dairy Products in Human Nutrition: Production, Composition and Health2013:390-411.

Livingstone KM, Lovegrove JA, Givens DI. The impact of substituting SFA in dairy products with MUFA or PUFA on CVD risk: evidence from human intervention studies. Nutrition research reviews. Dec 2012;25(2):193-206.

Maghsoudi Z, Ghiasvand R, Salehi-Abargouei A. Empirically derived dietary patterns and incident type 2 diabetes mellitus: a systematic review and meta-analysis on prospective observational studies. Public health nutrition. 2015.

Markey O, Vasilopoulou D, Givens DI, Lovegrove JA. Dairy and cardiovascular health: Friend or foe? Nutrition Bulletin. 2014;39(2):161-171.

Mascitelli L, Pezzetta F. Dairy intake, reduced body iron stores, and the development of type 2 diabetes mellitus. Archives of internal medicine. Nov 14 2005;165(20):2433-2434; author reply 2435.

Massey LK. Dairy food consumption, blood pressure and stroke. The Journal of nutrition. Jul 2001;131(7):1875-1878.

McDougall J. Dairy and type 2 diabetes mellitus: wrong conclusions. Archives of internal medicine. Nov 14 2005;165(20):2434; author reply 2435.

Misra A, Singhal N, Khurana L. Obesity, the metabolic syndrome, and type 2 diabetes in developing countries: role of dietary fats and oils. Journal of the American College of Nutrition. Jun 2010;29(3 Suppl):289s-301s.

Mizoue T, Yamaji T, Tabata S, et al. Dietary patterns and glucose tolerance abnormalities in Japanese men. The Journal of nutrition. May 2006;136(5):1352-1358.

Mozaffarian D, Clarke R. Quantitative effects on cardiovascular risk factors and coronary heart disease risk of replacing partially hydrogenated vegetable oils with other fats and oils. European journal of clinical nutrition. May 2009;63 Suppl 2:S22-33.

Oliveira A, Lopes C, Rodriguez-Artalejo F. Adherence to the Southern European Atlantic Diet and occurrence of nonfatal acute myocardial infarction. The American journal of clinical nutrition. Jul 2010;92(1):211-217.

Panagiotakos D, Bountziouka V, Zeimbekis A, Vlachou I, Polychronopoulos E. Food pattern analysis and prevalence of cardiovascular disease risk factors among elderly people from Mediterranean islands. Journal of medicinal food. Dec 2007;10(4):615-621.

Panagiotakos DB, Pitsavos C, Arvaniti F, Stefanadis C. Adherence to the Mediterranean food pattern predicts the prevalence of hypertension, hypercholesterolemia, diabetes and obesity, among healthy adults; the accuracy of the MedDietScore. Preventive medicine. Apr 2007;44(4):335-340.

Rasmussen O, Lauszus FF, Christiansen C, Thomsen C, Hermansen K. Differential effects of saturated and monounsaturated fat on blood glucose and insulin responses in subjects with non-insulin-dependent diabetes mellitus. The American journal of clinical nutrition. Feb 1996;63(2):249-253.

Rees K, Hartley L, Flowers N, et al. 'Mediterranean' dietary pattern for the primary prevention of cardiovascular disease. The Cochrane database of systematic reviews. 2013;8:Cd009825.

Safarian M, Shojaeizadeh MS, Ghayour-Mobarhan M, Esmailie H, Nematy M, Razavi A. Investigation of dietary patterns, healthy eating index and traditional risk factors of cardiovascular disease in 35-65 years old adults of Mashhad. Medical Journal of Mashhad University of Medical Sciences. 2013;56(Mashhad University of Medical Sciences, Mashhad, Iran.):226-235.

Segall J. Communicable disease associated with milk and dairy products. British medical journal (Clinical research ed.). Aug 21 1982;285(6341):575.

Silbernagel G, Fauler G, Renner W, et al. The relationships of cholesterol metabolism and plasma plant sterols with the severity of coronary artery disease. Journal of lipid research. Feb 2009;50(2):334-341.

Swain JF, McCarron PB, Hamilton EF, Sacks FM, Appel LJ. Characteristics of the diet patterns tested in the optimal macronutrient intake trial to prevent heart disease (OmniHeart): options for a heart-healthy diet. Journal of the American Dietetic Association. Feb 2008;108(2):257-265.

Tardy AL, Morio B, Chardigny JM, Malpuech-Brugere C. Ruminant and industrial sources of trans-fat and cardiovascular and diabetic diseases. Nutrition research reviews. Jun 2011;24(1):111-117.

Teunissen-Beekman KFM, van Baak MA. The role of dietary protein in blood pressure regulation. Current opinion in lipidology. Feb 2013;24(1):65-70.

Tholstrup T. Dairy products and cardiovascular disease. Current opinion in lipidology. Feb 2006;17(1):1-10.

Tillotson JL, Bartsch GE, Gorder D, Grandits GA, Stamler J. Food group and nutrient intakes at baseline in the Multiple Risk Factor Intervention Trial. The American journal of clinical nutrition. Jan 1997;65(1 Suppl):228s-257s.

Tong X, Dong JY, Wu ZW, Li W, Qin LQ. Dairy consumption and risk of type 2 diabetes mellitus: a meta-analysis of cohort studies. European journal of clinical nutrition. Sep 2011;65(9):1027-1031.

Turner KM, Keogh JB, Clifton PM. Dairy consumption and insulin sensitivity: A systematic review of short- and long-term intervention studies. Nutrition, Metabolism and Cardiovascular Diseases. 2014.

van Heyningen C. Cholesterol lowering margarine may not be useful in healthy fat modified diet. BMJ (Clinical research ed.). Jul 17 1999;319(7203):186.

Vos E, Cunnane SC, Lanzmann-Petithory D. n-3 fatty acids and cardiovascular events. New England Journal of Medicine. 2011;364(9):880-881.

Weggemans RM, Rudrum M, Trautwein EA. Intake of ruminant versus industrial trans fatty acids and risk of coronary heart disease - what is the evidence? European Journal of Lipid Science and Technology. Jun 2004;106(6):390-397.

Weisburger JH. Coffee, cream, and coronary heart disease. Epidemiology (Cambridge, Mass.). Mar 1994;5(2):263-264.

Willett WC. Dietary fats and coronary heart disease. Journal of internal medicine. Jul 2012;272(1):13-24.

Wylie-Rosett J. Dairy products and metabolic risk factors: How much do we know? Diabetes care. 2011;34(4):1064-1065.

Zatonski WA, McMichael AJ, Powles JW. Ecological study of reasons for sharp decline in mortality from ischaemic heart disease in Poland since 1991. BMJ (Clinical research ed.). Apr 4 1998;316(7137):1047-1051.

**Did not provide data on the relevant exposure of interest (n=51)**

Aune D, Norat T, Romundstad P, Vatten LJ. Dairy products and the risk of type 2 diabetes: a systematic review and dose-response meta-analysis of cohort studies. The American journal of clinical nutrition. Oct 2013;98(4):1066-1083.

Chen M, Sun Q, Giovannucci E, et al. Dairy consumption and risk of type 2 diabetes: 3 cohorts of US adults and an updated meta-analysis. BMC medicine. 2014;12:215.

Hu D, Huang J, Wang Y, Zhang D, Qu Y. Dairy foods and risk of stroke: a meta-analysis of prospective cohort studies. Nutrition, metabolism, and cardiovascular diseases : NMCD. May 2014;24(5):460-469.

O'Sullivan TA, Hafekost K, Mitrou F, Lawrence D. Food sources of saturated fat and the association with mortality: a meta-analysis. American journal of public health. Sep 2013;103(9):e31-42.

O'Sullivan TA, Hafekost K, Mitrou F, Lawrence D. Food sources of saturated fat and the association with mortality: a meta-analysis. American journal of public health. Sep 2013;103(9):e31-42.

Qin LQ, Xu JY, Han SF, Zhang ZL, Zhao YY, Szeto IM. Dairy consumption and risk of cardiovascular disease: an updated meta-analysis of prospective cohort studies. Asia Pacific journal of clinical nutrition. 2015;24(1):90-100.

Akter S, Kurotani K, Nanri A, et al. Dairy consumption is associated with decreased insulin resistance among the Japanese. Nutr. Res. Apr 2013;33(4):286-292.

Astrup A. Yogurt and dairy product consumption to prevent cardiometabolic diseases: epidemiologic and experimental studies. The American journal of clinical nutrition. May 2014;99(5 Suppl):1235s-1242s.

Bamia C, Orfanos P, Ferrari P, et al. Dietary patterns among older Europeans: the EPIC-Elderly study. British Journal of Nutrition. Jul 2005;94(1):100-113.

Bilenko N, Fraser D, Vardi H, Shai I, Shahar DR. Mediterranean diet and cardiovascular diseases in an Israeli population. Preventive medicine. Mar 2005;40(3):299-305.

Candido FG, Ton WT, Alfenas Rde C. Dairy products consumption versus type 2 diabetes prevention and treatment; a review of recent findings from human studies. Nutricion hospitalaria. Sep-Oct 2013;28(5):1384-1395.

Clifton PM. Protein and coronary heart disease: the role of different protein sources. Current atherosclerosis reports. Dec 2011;13(6):493-498.

Correa-Matos NJ, Vaghefi SB. Dairy Foods and Cardiovascular Diseases. Bioactive Food as Dietary Interventions for Cardiovascular Disease2013:319-332.

de Lorgeril M, Salen P. The Mediterranean-style diet for the prevention of cardiovascular diseases. Public health nutrition. Feb 2006;9(1a):118-123.

Delavar MA, Lye MS, Khor GL, Hassan S, Hanachi P. Dietary patterns and the metabolic syndrome in middle aged women, Babol, Iran. Asia Pacific journal of clinical nutrition. 2009;18(2):285-292.

DiBello JR, Kraft P, McGarvey ST, Goldberg R, Campos H, Baylin A. Comparison of 3 methods for identifying dietary patterns associated with risk of disease. American journal of epidemiology. Dec 15 2008;168(12):1433-1443.

Ellis IH. Communicable disease associated with milk and dairy products. British medical journal (Clinical research ed.). Sep 25 1982;285(6345):895.

Elmstahl S, Holmqvist O, Gullberg B, Johansson U, Berglund G. Dietary patterns in high and low consumers of meat in a Swedish cohort study. Appetite. Apr 1999;32(2):191-206.

Elwood PC, Givens DI, Beswick AD, Fehily AM, Pickering JE, Gallacher J. The survival advantage of milk and dairy consumption: an overview of evidence from cohort studies of vascular diseases, diabetes and cancer. Journal of the American College of Nutrition. Dec 2008;27(6):723s-734s.

Ford DW, Jensen GL, Hartman TJ, Wray L, Smiciklas-Wright H. Association between dietary quality and mortality in older adults: a review of the epidemiological evidence. Journal of nutrition in gerontology and geriatrics. 2013;32(2):85-105.

Fraser G. Commentary: Protection from stroke by eating animal foods? Surely not! International journal of epidemiology. Aug 2003;32(4):543-545.

Gao D, Ning N, Wang C, et al. Dairy products consumption and risk of type 2 diabetes: systematic review and dose-response meta-analysis. PloS one. 2013;8(9):e73965.

Gayet-Boyer C, Tenenhaus-Aziza F, Prunet C, et al. Is there a linear relationship between the dose of ruminant trans-fatty acids and cardiovascular risk markers in healthy subjects: results from a systematic review and meta-regression of randomised clinical trials. British Journal of Nutrition. Dec 2014;112(12):1914-1922.

Gebauer SK, Chardigny JM, Jakobsen MU, et al. Effects of ruminant trans fatty acids on cardiovascular disease and cancer: a comprehensive review of epidemiological, clinical, and mechanistic studies. Advances in nutrition (Bethesda, Md.). Jul 2011;2(4):332-354.

Gibson RA, Makrides M, Smithers LG, Voevodin M, Sinclair AJ. The effect of dairy foods on CHD: a systematic review of prospective cohort studies. The British journal of nutrition. Nov 2009;102(9):1267-1275.

Hirahatake KM, Slavin JL, Maki KC, Adams SH. Associations between dairy foods, diabetes, and metabolic health: potential mechanisms and future directions. Metabolism: clinical and experimental. May 2014;63(5):618-627.

Hu D, Huang J, Wang Y, Zhang D, Qu Y. Dairy foods and risk of stroke: a meta-analysis of prospective cohort studies. Nutrition, metabolism, and cardiovascular diseases : NMCD. May 2014;24(5):460-469.

Huth PJ, Park KM. Influence of dairy product and milk fat consumption on cardiovascular disease risk: a review of the evidence. Advances in nutrition (Bethesda, Md.). May 2012;3(3):266-285.

Ismail J, Jafar TH, Jafary FH, White F, Faruqui AM, Chaturvedi N. Risk factors for non-fatal myocardial infarction in young South Asian adults. Heart (British Cardiac Society). Mar 2004;90(3):259-263.

Jakobsen MU, Bysted A, Andersen NL, et al. Intake of ruminant trans fatty acids and risk of coronary heart disease - An overview. Atheroscler. Suppl. May 2006;7(2):9-11.

Kontogianni MD, Panagiotakos DB, Chrysohoou C, Pitsavos C, Stefanadis C. Modelling dairy intake on the development of acute coronary syndromes: the CARDIO2000 study. European journal of cardiovascular prevention and rehabilitation : official journal of the European Society of Cardiology, Working Groups on Epidemiology & Prevention and Cardiac Rehabilitation and Exercise Physiology. Oct 2006;13(5):791-797.

Kratz M, Baars T, Guyenet S. The relationship between high-fat dairy consumption and obesity, cardiovascular, and metabolic disease. European journal of nutrition. Feb 2013;52(1):1-24.

Kwak HS, Ganesan P, Mijan MA. Butter, Ghee, and Cream Products. Milk and Dairy Products in Human Nutrition: Production, Composition and Health2013:390-411.

Livingstone KM, Lovegrove JA, Givens DI. The impact of substituting SFA in dairy products with MUFA or PUFA on CVD risk: evidence from human intervention studies. Nutrition research reviews. Dec 2012;25(2):193-206.

Maghsoudi Z, Ghiasvand R, Salehi-Abargouei A. Empirically derived dietary patterns and incident type 2 diabetes mellitus: a systematic review and meta-analysis on prospective observational studies. Public health nutrition. 2015.

Markey O, Vasilopoulou D, Givens DI, Lovegrove JA. Dairy and cardiovascular health: Friend or foe? Nutrition Bulletin. 2014;39(2):161-171.

Mascitelli L, Pezzetta F. Dairy intake, reduced body iron stores, and the development of type 2 diabetes mellitus. Archives of internal medicine. Nov 14 2005;165(20):2433-2434; author reply 2435.

Massey LK. Dairy food consumption, blood pressure and stroke. The Journal of nutrition. Jul 2001;131(7):1875-1878.

McDougall J. Dairy and type 2 diabetes mellitus: wrong conclusions. Archives of internal medicine. Nov 14 2005;165(20):2434; author reply 2435.

Misra A, Singhal N, Khurana L. Obesity, the metabolic syndrome, and type 2 diabetes in developing countries: role of dietary fats and oils. Journal of the American College of Nutrition. Jun 2010;29(3 Suppl):289s-301s.

Mizoue T, Yamaji T, Tabata S, et al. Dietary patterns and glucose tolerance abnormalities in Japanese men. The Journal of nutrition. May 2006;136(5):1352-1358.

Mozaffarian D, Clarke R. Quantitative effects on cardiovascular risk factors and coronary heart disease risk of replacing partially hydrogenated vegetable oils with other fats and oils. European journal of clinical nutrition. May 2009;63 Suppl 2:S22-33.

Oliveira A, Lopes C, Rodriguez-Artalejo F. Adherence to the Southern European Atlantic Diet and occurrence of nonfatal acute myocardial infarction. The American journal of clinical nutrition. Jul 2010;92(1):211-217.

Panagiotakos D, Bountziouka V, Zeimbekis A, Vlachou I, Polychronopoulos E. Food pattern analysis and prevalence of cardiovascular disease risk factors among elderly people from Mediterranean islands. Journal of medicinal food. Dec 2007;10(4):615-621.

Panagiotakos DB, Pitsavos C, Arvaniti F, Stefanadis C. Adherence to the Mediterranean food pattern predicts the prevalence of hypertension, hypercholesterolemia, diabetes and obesity, among healthy adults; the accuracy of the MedDietScore. Preventive medicine. Apr 2007;44(4):335-340.

Rasmussen O, Lauszus FF, Christiansen C, Thomsen C, Hermansen K. Differential effects of saturated and monounsaturated fat on blood glucose and insulin responses in subjects with non-insulin-dependent diabetes mellitus. The American journal of clinical nutrition. Feb 1996;63(2):249-253.

Rees K, Hartley L, Flowers N, et al. 'Mediterranean' dietary pattern for the primary prevention of cardiovascular disease. The Cochrane database of systematic reviews. 2013;8:Cd009825.

Safarian M, Shojaeizadeh MS, Ghayour-Mobarhan M, Esmailie H, Nematy M, Razavi A. Investigation of dietary patterns, healthy eating index and traditional risk factors of cardiovascular disease in 35-65 years old adults of Mashhad. Medical Journal of Mashhad University of Medical Sciences. 2013;56(Mashhad University of Medical Sciences, Mashhad, Iran.):226-235.

Segall J. Communicable disease associated with milk and dairy products. British medical journal (Clinical research ed.). Aug 21 1982;285(6341):575.

Silbernagel G, Fauler G, Renner W, et al. The relationships of cholesterol metabolism and plasma plant sterols with the severity of coronary artery disease. Journal of lipid research. Feb 2009;50(2):334-341.

Swain JF, McCarron PB, Hamilton EF, Sacks FM, Appel LJ. Characteristics of the diet patterns tested in the optimal macronutrient intake trial to prevent heart disease (OmniHeart): options for a heart-healthy diet. Journal of the American Dietetic Association. Feb 2008;108(2):257-265.

Tardy AL, Morio B, Chardigny JM, Malpuech-Brugere C. Ruminant and industrial sources of trans-fat and cardiovascular and diabetic diseases. Nutrition research reviews. Jun 2011;24(1):111-117.

Teunissen-Beekman KFM, van Baak MA. The role of dietary protein in blood pressure regulation. Current opinion in lipidology. Feb 2013;24(1):65-70.

Tholstrup T. Dairy products and cardiovascular disease. Current opinion in lipidology. Feb 2006;17(1):1-10.

Tillotson JL, Bartsch GE, Gorder D, Grandits GA, Stamler J. Food group and nutrient intakes at baseline in the Multiple Risk Factor Intervention Trial. The American journal of clinical nutrition. Jan 1997;65(1 Suppl):228s-257s.

Tong X, Dong JY, Wu ZW, Li W, Qin LQ. Dairy consumption and risk of type 2 diabetes mellitus: a meta-analysis of cohort studies. European journal of clinical nutrition. Sep 2011;65(9):1027-1031.

Turner KM, Keogh JB, Clifton PM. Dairy consumption and insulin sensitivity: A systematic review of short- and long-term intervention studies. Nutrition, Metabolism and Cardiovascular Diseases. 2014.

van Heyningen C. Cholesterol lowering margarine may not be useful in healthy fat modified diet. BMJ (Clinical research ed.). Jul 17 1999;319(7203):186.

Vos E, Cunnane SC, Lanzmann-Petithory D. n-3 fatty acids and cardiovascular events. New England Journal of Medicine. 2011;364(9):880-881.

Weggemans RM, Rudrum M, Trautwein EA. Intake of ruminant versus industrial trans fatty acids and risk of coronary heart disease - what is the evidence? European Journal of Lipid Science and Technology. Jun 2004;106(6):390-397.

Weisburger JH. Coffee, cream, and coronary heart disease. Epidemiology (Cambridge, Mass.). Mar 1994;5(2):263-264.

Willett WC. Dietary fats and coronary heart disease. Journal of internal medicine. Jul 2012;272(1):13-24.

Wylie-Rosett J. Dairy products and metabolic risk factors: How much do we know? Diabetes care. 2011;34(4):1064-1065.

Zatonski WA, McMichael AJ, Powles JW. Ecological study of reasons for sharp decline in mortality from ischaemic heart disease in Poland since 1991. BMJ (Clinical research ed.). Apr 4 1998;316(7137):1047-1051.

**Only provided data on dietary patterns (n=38)**

Abiemo EE, Alonso A, Nettleton JA, et al. Relationships of the Mediterranean dietary pattern with insulin resistance and diabetes incidence in the Multi-Ethnic Study of Atherosclerosis (MESA). The British journal of nutrition. Apr 28 2013;109(8):1490-1497.

Anderson AL, Harris TB, Tylavsky FA, et al. Dietary patterns and survival of older adults. Journal of the American Dietetic Association. Jan 2011;111(1):84-91.

Bernstein AM, Sun Q, Hu FB, Stampfer MJ, Manson JE, Willett WC. Major dietary protein sources and risk of coronary heart disease in women. Circulation. Aug 31 2010;122(9):876-883.

Bonthuis M, Hughes MC, Ibiebele TI, Green AC, van der Pols JC. Dairy consumption and patterns of mortality of Australian adults. European journal of clinical nutrition. Jun 2010;64(6):569-577.

Brunner EJ, Mosdol A, Witte DR, et al. Dietary patterns and 15-y risks of major coronary events, diabetes, and mortality. The American journal of clinical nutrition. May 2008;87(5):1414-1421.

Choi HK, Willett WC, Stampfer MJ, Rimm E, Hu FB. Dairy consumption and risk of type 2 diabetes mellitus in men: a prospective study. Archives of internal medicine. May 9 2005;165(9):997-1003.

Drogan D, Hoffmann K, Schulz M, Bergmann MM, Boeing H, Weikert C. A food pattern predicting prospective weight change is associated with risk of fatal but not with nonfatal cardiovascular disease. The Journal of nutrition. Aug 2007;137(8):1961-1967.

Elwood PC, Pickering JE, Fehily AM. Milk and dairy consumption, diabetes and the metabolic syndrome: the Caerphilly prospective study. Journal of epidemiology and community health. Aug 2007;61(8):695-698.

Fehily AM, Yarnell JW, Sweetnam PM, Elwood PC. Diet and incident ischaemic heart disease: the Caerphilly Study. The British journal of nutrition. Mar 1993;69(2):303-314.

Fumeron F, Lamri A, Emery N, et al. Dairy products and the metabolic syndrome in a prospective study, DESIR. Journal of the American College of Nutrition. Oct 2011;30(5 Suppl 1):454s-463s.

He K, Merchant A, Rimm EB, et al. Dietary fat intake and risk of stroke in male US healthcare professionals: 14 year prospective cohort study. BMJ (Clinical research ed.). Oct 4 2003;327(7418):777-782.

Hodge AM, English DR, O'Dea K, Giles GG. Dietary patterns and diabetes incidence in the Melbourne Collaborative Cohort Study. American journal of epidemiology. Mar 15 2007;165(6):603-610.

Hoffmann K, Boeing H, Boffetta P, et al. Comparison of two statistical approaches to predict all-cause mortality by dietary patterns in German elderly subjects. The British journal of nutrition. May 2005;93(5):709-716.

Hoffmann K, Zyriax BC, Boeing H, Windler E. A dietary pattern derived to explain biomarker variation is strongly associated with the risk of coronary artery disease. The American journal of clinical nutrition. Sep 2004;80(3):633-640.

Holmberg S, Thelin A, Stiernstrom EL. Food choices and coronary heart disease: a population based cohort study of rural Swedish men with 12 years of follow-up. International journal of environmental research and public health. Oct 2009;6(10):2626-2638.

Hsiao PY, Mitchell DC, Coffman DL, et al. Dietary patterns and relationship to obesity-related health outcomes and mortality in adults 75 years of age or greater. The journal of nutrition, health & aging. 2013;17(6):566-572.

Hu FB, Rimm EB, Stampfer MJ, Ascherio A, Spiegelman D, Willett WC. Prospective study of major dietary patterns and risk of coronary heart disease in men. The American journal of clinical nutrition. Oct 2000;72(4):912-921.

Hu FB, Stampfer MJ, Manson JE, et al. Dietary saturated fats and their food sources in relation to the risk of coronary heart disease in women. The American journal of clinical nutrition. Dec 1999;70(6):1001-1008.

Kant AK, Graubard BI, Schatzkin A. Dietary patterns predict mortality in a national cohort: the National Health Interview Surveys, 1987 and 1992. The Journal of nutrition. Jul 2004;134(7):1793-1799.

Kant AK, Leitzmann MF, Park Y, Hollenbeck A, Schatzkin A. Patterns of recommended dietary behaviors predict subsequent risk of mortality in a large cohort of men and women in the United States. The Journal of nutrition. Jul 2009;139(7):1374-1380.

Liu S, Choi HK, Ford E, et al. A prospective study of dairy intake and the risk of type 2 diabetes in women. Diabetes care. Jul 2006;29(7):1579-1584.

Margolis KL, Wei F, de Boer IH, et al. A diet high in low-fat dairy products lowers diabetes risk in postmenopausal women. The Journal of nutrition. Nov 2011;141(11):1969-1974.

Martínez-González MA, Zazpe I, Razquin C, et al. Empirically-derived food patterns and the risk of total mortality and cardiovascular events in the PREDIMED study. Clinical Nutrition. 2015.

Maruyama K, Iso H, Date C, et al. Dietary patterns and risk of cardiovascular deaths among middle-aged Japanese: JACC Study. Nutrition, metabolism, and cardiovascular diseases : NMCD. Jun 2013;23(6):519-527.

Masala G, Ceroti M, Pala V, et al. A dietary pattern rich in olive oil and raw vegetables is associated with lower mortality in Italian elderly subjects. The British journal of nutrition. Aug 2007;98(2):406-415.

Meyer J, Doring A, Herder C, Roden M, Koenig W, Thorand B. Dietary patterns, subclinical inflammation, incident coronary heart disease and mortality in middle-aged men from the MONICA/KORA Augsburg cohort study. European journal of clinical nutrition. Jul 2011;65(7):800-807.

Montonen J, Knekt P, Harkanen T, et al. Dietary patterns and the incidence of type 2 diabetes. American journal of epidemiology. Feb 1 2005;161(3):219-227.

O'Connor LM, Lentjes MA, Luben RN, Khaw KT, Wareham NJ, Forouhi NG. Dietary dairy product intake and incident type 2 diabetes: a prospective study using dietary data from a 7-day food diary. Diabetologia. May 2014;57(5):909-917.

Osler M, Heitmann BL, Gerdes LU, Jorgensen LM, Schroll M. Dietary patterns and mortality in Danish men and women: a prospective observational study. The British journal of nutrition. Feb 2001;85(2):219-225.

Osler M, Helms Andreasen A, Heitmann B, et al. Food intake patterns and risk of coronary heart disease: a prospective cohort study examining the use of traditional scoring techniques. European journal of clinical nutrition. Jul 2002;56(7):568-574.

Panagiotakos D, Pitsavos C, Chrysohoou C, et al. Dietary patterns and 5-year incidence of cardiovascular disease: a multivariate analysis of the ATTICA study. Nutrition, metabolism, and cardiovascular diseases : NMCD. May 2009;19(4):253-263.

Shimazu T, Kuriyama S, Hozawa A, et al. Dietary patterns and cardiovascular disease mortality in Japan: a prospective cohort study. International journal of epidemiology. Jun 2007;36(3):600-609.

Shuster GF. Commentary: Dietary patterns including fruits, vegetables, whole grains, low fat dairy, and lean meats lowered mortality risk in women. Evidence-Based Nursing. 2000;3(4):130.

van Dam RM, Rimm EB, Willett WC, Stampfer MJ, Hu FB. Dietary patterns and risk for type 2 diabetes mellitus in U.S. men. Annals of internal medicine. Feb 5 2002;136(3):201-209.

Villegas R, Yang G, Gao YT, et al. Dietary patterns are associated with lower incidence of type 2 diabetes in middle-aged women: the Shanghai Women's Health Study. International journal of epidemiology. Jun 2010;39(3):889-899.

Waijers PM, Ocke MC, van Rossum CT, et al. Dietary patterns and survival in older Dutch women. The American journal of clinical nutrition. May 2006;83(5):1170-1176.

Warensjo E, Jansson JH, Cederholm T, et al. Biomarkers of milk fat and the risk of myocardial infarction in men and women: a prospective, matched case-control study. The American journal of clinical nutrition. Jul 2010;92(1):194-202.

Willett WC, Stampfer MJ, Manson JE, et al. Intake of trans fatty acids and risk of coronary heart disease among women. Lancet. Mar 6 1993;341(8845):581-585.

**Did not provide data on the relevant outcome of interest (n=13)**

Lako JV, Nguyen VC. Dietary patterns and risk factors of diabetes mellitus among urban indigenous women in Fiji. Asia Pacific journal of clinical nutrition. 2001;10(3):188-193.

Jensen J, Bysted A, Dawids S, Hermansen K, Holmer G. The effect of palm oil, lard, and puff-pastry margarine on postprandial lipid and hormone responses in normal-weight and obese young women. British Journal of Nutrition. Dec 1999;82(6):469-479.

Judd JT, Baer DJ, Clevidence BA, et al. Effects of margarine compared with those of butter on blood lipid profiles related to cardiovascular disease risk factors in normolipemic adults fed controlled diets. The American journal of clinical nutrition. Oct 1998;68(4):768-777.

Lacroix E, Charest A, Cyr A, et al. Randomized controlled study of the effect of a butter naturally enriched in trans fatty acids on blood lipids in healthy women. The American journal of clinical nutrition. Feb 2012;95(2):318-325.

Livingstone KM, Lovegrove JA, Cockcroft JR, Elwood PC, Pickering JE, Givens DI. Does Dairy Food Intake Predict Arterial Stiffness and Blood Pressure in Men? Evidence from the Caerphilly Prospective Study. Hypertension. Jan 2013;61(1):42-+.

Matsumoto M, Ishikawa S, Nakamura Y, Kayaba K, Kajii E. Consumption of dairy products and cancer risks. Journal of epidemiology / Japan Epidemiological Association. Mar 2007;17(2):38-44.

McNaughton SA, Mishra GD, Stephen AM, Wadsworth MEJ. Dietary patterns throughout adult life are associated with body mass index, waist circumference, blood pressure, and red cell folate. J. Nutr. Jan 2007;137(1):99-105.

Moon HK, Kim CY, Lee SW. Exploratory correlations of dietary nutrients with prostate cancer mortality using over two decades of observations in Korea. Molecular nutrition & food research. Feb 2009;53(2):185-190.

Moore TJ, Vollmer WM, Appel LJ, et al. Effect of dietary patterns on ambulatory blood pressure : results from the Dietary Approaches to Stop Hypertension (DASH) Trial. DASH Collaborative Research Group. Hypertension. Sep 1999;34(3):472-477.

Pereira MA, Jacobs DR, Jr., Van Horn L, Slattery ML, Kartashov AI, Ludwig DS. Dairy consumption, obesity, and the insulin resistance syndrome in young adults: the CARDIA Study. Jama. Apr 24 2002;287(16):2081-2089.

Sakauchi F, Mori M, Washio M, et al. Dietary habits and risk of urothelial cancer death in a large-scale cohort study (JACC Study) in Japan. Nutrition and cancer. 2004;50(1):33-39.

Toledo E, Delgado-Rodriguez M, Estruch R, et al. Low-fat dairy products and blood pressure: follow-up of 2290 older persons at high cardiovascular risk participating in the PREDIMED study. The British journal of nutrition. Jan 2009;101(1):59-67.

Yannakoulia M, Yiannakouris N, Melistas L, Kontogianni MD, Malagaris I, Mantzoros CS. A dietary pattern characterized by high consumption of whole-grain cereals and low-fat dairy products and low consumption of refined cereals is positively associated with plasma adiponectin levels in healthy women. Metab.-Clin. Exp. Jun 2008;57(6):824-830.

**Provided duplicate data (n=5)**

Bailey BW, Smith BK, Sullivan DK, Donnelly JE. The Effects of Dairy Consumption on Metabolic Risk Factors For Ischemic Heart Disease. Obesity. Nov 2011;19:S112-S113.

Otto MCD, Nettleton JA, Lemaitre RN, et al. Biomarkers of Dairy Fatty Acids and Risk of Cardiovascular Disease in the Multi-Ethnic Study of Atherosclerosis. Journal of the American Heart Association. Aug 2013;2(4):14.

Praagman J, Franco OH, Ikram MA, et al. Dairy products and the risk of stroke and coronary heart disease: the Rotterdam Study. European journal of nutrition. 2014.

Elwood PC, Pickering JE, Givens DI, Gallacher JE. The Consumption of Milk and Dairy Foods and the Incidence of Vascular Disease and Diabetes: An Overview of the Evidence. Lipids. Oct 2010;45(10):925-939.

Elwood PC, Pickering JE, Hughes J, Fehily AM, Ness AR. Milk drinking, ischaemic heart disease and ischaemic stroke II. Evidence from cohort studies. European journal of clinical nutrition. May 2004;58(5):718-724.
