# Supplementary material for: Is Butter Back? A Systematic Review and Meta-Analysis of Butter Consumption and Risk of Cardiovascular Disease, Diabetes, and Total Mortality
Source: PLoS One. 2016 Jun 29;11(6):e0158118. doi: 10.1371/journal.pone.0158118 (PMC4927102; doi:10.1371/journal.pone.0158118)
Supplement: S5 File — (DOCX) [file pone.0158118.s007.docx]

**S5 File. Supporting Information. Standardized estimation strategies for missing data**

When missing information on median exposure, number of participants or person-years, and number of events in any category of exposure could not be obtained by direct author contact, we used a standard approach to estimate these values. We estimated median intake by using the midpoint for each category; when the highest category was open-ended, we assumed the category range to be the same as the adjacent category, if the lowest category of intake was open-ended, we used the midpoint between the upper bound and zero as the median intake. Total person-years of follow-up was estimated, when not reported, by multiplying the total number of participants by the median follow-up duration; or by half of the maximum follow-up if the median was not reported. When the distribution of person-years in each category was not reported, we estimated it to be distributed evenly by the percentage of participants in each category.
